# The Behavioral, Emotional, and Social Skills Inventory (BESSI): Psychometric Properties of a German-Language Adaptation, Temporal Stabilities of the Skills, and Associations with Personality and Intelligence

**DOI:** 10.3390/jintelligence10030063

**Published:** 2022-09-05

**Authors:** Clemens M. Lechner, Thomas Knopf, Christopher M. Napolitano, Beatrice Rammstedt, Brent W. Roberts, Christopher J. Soto, Marion Spengler

**Affiliations:** 1 Department Survey Design and Methodology, GESIS—Leibniz Institute for the Social Sciences, P.O. Box 12 21 55, 68072 Mannheim, Germany; 2 College of Education, University of Illinois Urbana-Champaign, 1310 S. Sixth St., Champaign, IL 61820, USA; 3 Department of Psychology, College of Liberal Arts & Sciences, University of Illinois Urbana-Champaign, 308 Psychology Bldg, MC-716 603 East Daniel St., Champaign, IL 61820, USA; 4 Psychology Department, Colby College, 5550 Mayflower Hill, Waterville, MN 04901, USA; 5 Hochschule für Gesundheit und Medizin, MSB Medical School Berlin, Rüdesheimer Straße 50, 14197 Berlin, Germany

**Keywords:** non-cognitive skills, socio-emotional skills, assessment

## Abstract

Social, emotional, and behavioral (SEB) skills comprise a broad set of abilities that are essential for building and maintaining relationships, regulating emotions, selecting and pursuing goals, or exploring novel stimuli. Toward an improved SEB skill assessment, Soto and colleagues recently introduced the Behavioral, Emotional, and Social Skills Inventory (BESSI). Measuring 32 facets from 5 domains with 192 items (assessment duration: ~15 min), BESSI constitutes the most extensive SEB inventory to date. However, so far, BESSI exists only in English. In three studies, we comprehensively validated a novel German-language adaptation, BESSI-G. Moreover, we expanded evidence on BESSI in three ways by (1) assessing the psychometric properties of the 32 individual skill facets, in addition to their domain-level structure; (2) providing first insights into the temporal stabilities of the 32 facets over 1.5 and 8 months; and (3) investigating the domains’ and facets’ associations with intelligence, in addition to personality traits. Results show that BESSI-G exhibits good psychometric properties (unidimensionality, reliability, factorial validity). Its domain-level structure is highly similar to that of the English-language source version. The facets show high temporal stabilities, convergent validity with personality traits, and discriminant validity with fluid and crystallized intelligence. We discuss implications for research on SEB skills.

## 1. Introduction

Social, emotional, and behavioral (SEB) skills denote people’s capacity to build and maintain social relationships, regulate emotions, and manage goal- and learning-directed behaviors (Abrahams et al. 2019; Schoon 2021; Soto et al. 2022). SEB skills comprise a broad set of inter- and intrapersonal abilities beyond those measured by traditional intelligence test that are sometimes referred to as “non-cognitive skills”, “soft skills” or “character skills”, although the term SEB skills is arguably more general and less value-laden. SEB skills, variously measured, predict educational achievement and attainment, job performance, well-being, health, and other consequential life outcomes—often above and beyond intelligence, which is traditionally seen as the major driver of many aspects of life success (e.g., Brandt et al. 2020; Napolitano et al. 2021; OECD 2015; Spengler et al. 2015).

Despite growing interest in SEB skills from researchers, policymakers, and practitioners, the assessment of SEB skills has long lagged behind that of intelligence. Absent a standard model of the structure and content of SEB skills, the field continues to be plagued by a proliferation of measures of varied scope and quality. Toward an improved assessment of SEB skills, Soto et al. (2022) recently introduced an integrative SEB skill framework and an attendant inventory: the Behavioral, Emotional, and Social Skills Inventory (BESSI). Synthesizing previous conceptualizations, BESSI offers what is arguably the most comprehensive and fine-grained framework for assessing SEB skills to date. Across multiple samples, BESSI showed promising psychometric properties in terms of reliability, factorial (structural) validity, as well as its nomological network and criterion validity.

The integrative assessment framework of BESSI holds promise to become a guidepost that helps advance research on SEB skills. However, so far, BESSI exists only in its original English-language version. In order to expand the scope of application of BESSI, we here present a German-language adaptation and further validation of BESSI. We comprehensively assess the psychometric properties of the German-language adaptation (henceforth “BESSI-G”) in three studies comprising multiple samples and assessment waves. Moreover, we expand evidence on BESSI more broadly in three ways: (1) By assessing the fit of the 32 individual skill facets that are the building blocks of BESSI, (2) providing first insights into the temporal stabilities of the 32 facets over 1.5 to 8 months, and (3) investigating these facets’ associations with intelligence (in addition to personality traits). The original study by Soto et al. (2022) focused on the fit of the full model comprising all skills and did not yet investigate the fit of individual skill facets. It also did not investigate their test–retest stabilities or associations with intelligence. Before presenting results from our three studies, we briefly review the BESSI assessment framework.

## 2. The Behavioral, Emotional, and Social Skills Inventory (BESSI)

**Figure 1:**
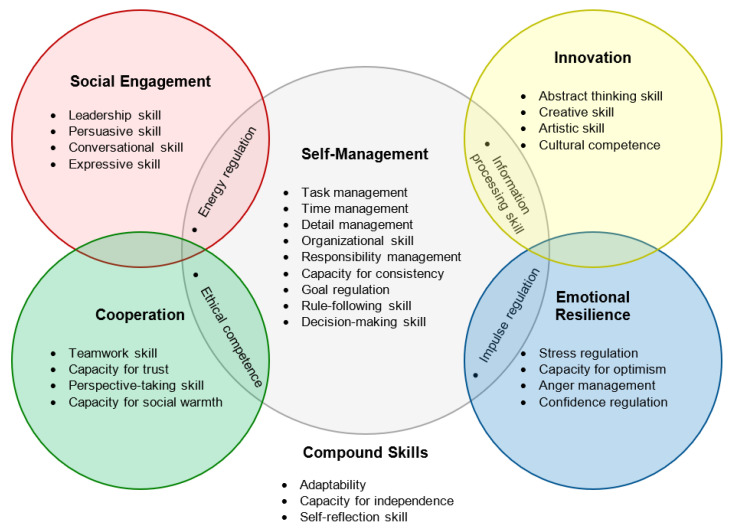
SEB skill domains and facets in the BESSI framework proposed by Soto et al. (2022).

Despite its vibrancy in recent years, research on SEB skills has long suffered from a high level of fragmentation, terminological confusion, and a lack of consensus regarding the definition of SEB skills as well as how to best assess them (Abrahams et al. 2019; Napolitano et al. 2021; Schoon 2021). With the aim to consolidate previous conceptualizations and taxonomies of SEB skills, Soto et al. (2022) (see also Napolitano et al. 2021), proposed an integrative framework for defining and organizing SEB skills. These authors defined SEB skills as functional capacities that relate to a person’s maximum ability to show SEB skill-related behaviors when the situation calls for it. Their hierarchical framework, shown in Figure 1, distinguishes between 32 SEB skill facets that are grouped in 5 broader skill domains (i.e., the colored circles in Figure 1): Social Engagement, Cooperation, Self-Management, Emotional Resilience, and Innovation.

Akin to other SEB skill assessment frameworks—for example, the OECD’s recent Study on Social and Emotional Skills (SSES; Chernyshenko et al. 2018)—the broader domains resemble the Big Five domains, which are the dominant framework in individual differences research. Soto et al. (2022) argued that the Big Five provide a comprehensive, and the empirically best-validated, framework to conceptualize individual differences in functional capacities as well as behaviors. Thus, the domain of Self-Management Skills is theoretically and empirically related to the Big Five domain Conscientiousness, Social Engagement Skills are related to the Big Five domain Extraversion, Cooperation is related to Agreeableness, Emotional Resilience is related to Neuroticism, and Innovation Skills are related to Openness. Most of the 32 individual SEB skills are uniquely assigned to one of these five domains. Some of the facets, labeled “interstitial facets” by Soto et al. (2022), fall in between two domains as per their loadings. Moreover, three facets, labeled “compound facets”, do not fall under any of the domains but add distinct content.

Along with this framework, Soto et al. (2022) introduced BESSI, a novel inventory to assess the SEB skills distinguished by their framework. Measuring each of the 32 SEB skill facets with 6 items (192 items in total), BESSI constitutes one of the most comprehensive SEB inventories to date. By grouping the 32 facets into 5 skill domains, BESSI allows for analyses both on the level of global domains and narrow facets. The option to analyze facets is a key asset because narrow facets add predictive power for life outcomes compared to domains, allow for a better understanding of mechanisms linking SEB skills to life outcomes, and offer a more apt target for interventions compared to global domains (e.g., Danner et al. 2021; Stewart et al. 2022). Global domains, meanwhile, offer a more parsimonious description and may be appropriate when assessment time and questionnaire space are limited, or when outcomes of interest are similarly global.

Crucially, although the organization of the five BESSI domains closely resembles the familiar five-factor structure from the realm of personality research, the response format of BESSI explicitly asks about perceived ability levels. That is, BESSI asks how well one can perform various tasks, rather than about typical behaviors (i.e., personality traits), in line with Soto et al.’s (2022) conceptualization of SEB skills as functional capacities. This allows for a clearer distinction between personality traits and SEB skills in a research field that has often used both concepts interchangeably (e.g., Lechner et al. 2019).

Across five studies and multiple adolescent and adult samples, BESSI showed good psychometric properties in Soto et al. (2022). Specifically, its facets showed high to very high internal consistencies, reached acceptable fit when modeled in a joint 32-facet item factor analysis (IFA) model, and clustered in the five broad domains largely as expected. Moreover, BESSI’s facets and domains showed convergent and discriminant validity in relation to the Big Five personality traits and a variety of other SEB skill measures. Importantly, BESSI’s domains and facets were also related to a wide range of outcomes including academic achievement and engagement, social relationships, and well-being—often incrementally over the Big Five. Although based on self-reports and cross-sectional data, these findings suggest that SEB skills measured with BESSI provide unique information in predicting life outcomes that is related to, yet distinct from, personality traits. These findings lend support to the idea that SEB skills can be meaningfully distinguished from personality traits.

## 3. Overview over the Present Research

In sum, BESSI provides a promising new tool for assessing SEB skills in a way that is valid, reliable, and comprehensive yet efficient. That said, Soto et al.’s (2022) initial studies were confined to English-speaking (mostly US) participants and the English-language source version of BESSI. For BESSI to be used in future research on SEB skills around the globe, additional language versions besides English are needed. To contribute to this endeavor, we therefore developed a German-language adaptation of BESSI, termed BESSI-G. Using this German-language adaptation, we set out to answer several fundamental questions about the SEB skills measured by BESSI, including their temporal stability and associations with intelligence.

Our validation of BESSI-G has three parts. In Study 1, a pilot study, we comprehensively assessed the psychometric properties of the initial translations of the 32 facet scales. In addition to internal consistency as an estimate of reliability, we assessed the facets’ test–retest correlation over 1.5 months, providing the first evidence on the temporal stability of the SEB skills assessed by BESSI. In Study 2, the main study, we assessed the same psychometric properties of a slightly revised second version of BESSI-G facet scales using data from a fresh adult sample. We also estimated the test–retest stabilities and true-score correlations over approximately 8 months to gauge the temporal stability of the BESSI facets over an extended period. Moreover, we test the domain-level structure of BESSI-G. Finally, in Study 3, we present evidence on the convergent and discriminant validity of BESSI-G’s facets and domains in relation to personality traits (as in the original study by Soto et al. 2022) and to fluid and crystallized intelligence, thus presenting first evidence on how BESSI(-G) relates to cognitive abilities.

## 4. Study 1: Pilot Study

The aim of Study 1 was to assess the psychometric properties of the initial version of the German-language adaptation of BESSI (BESSI-G v0.1). Whereas the original publication of the English-language source version of BESSI (Soto et al. 2022) focused on the joint structure of all 32 BESSI facets, in Study 1 we focused on the 32 individual skill facets as the building blocks of the newly translated inventory. We examined their (uni-)dimensionality, reliability (including test–retest stability), and factorial validity. Such facet-level analyses are important to determine the psychometric properties of the individual facets when using single items as input. These analyses are also informative if item parcels are later to be used as input for factor analyses, as Soto et al. (2022) did in their analyses of the original BESSI, because parcels require unidimensionality.

### 4.1. Method

#### 4.1.1. Data

Data for Study 1 came from 1164 adolescents and adults aged 14 to 64 years residing in Germany whose native language was German. We determined sample size based on simulation studies suggesting that samples sizes of at least 500, and sometimes 1000+, are needed to obtain stable correlations on the observed and latent-variable level (Kretzschmar and Gignac 2019; Schönbrodt and Perugini 2013). We collected the data via a commercial online access panel provider (Respondi AG). Respondents received a small monetary incentive for participation. For adults (20–64 years), there was a quota for age, gender, and education according to the German Microcensus 2017, ensuring that the sample was sufficiently diverse and resembled the general population in terms of its sociodemographic compositions. For teenagers (14–19 years), there was a quota for gender (quotas for age and education were not feasible). The data collection took place in January 2021. After carefully screening out 30 cases that provided invalid responses (e.g., straightliners), our final analysis sample for Study 1 comprised 1134 respondents.

To investigate test–retest stability and administer additional measures used in Study 3 (personality traits and intelligence), we invited a subset of the T0 sample to participate in up to three additional waves in February 2021 (T1, n = 727, focusing on intelligence), March 2021 (T2, n = 597, focusing on the BESSI-G retest and testing potential replacement items), and May 2021 (T3, n = 300, focusing on the retest of the potential replacement items).

#### 4.1.2. Materials

We translated BESSI from English to German using the TRAPD approach (Harkness 2003). TRAPD is a team-based translation approach that represents the current gold standard in questionnaire translation. It produces superior translations compared to traditional translation approaches such as backtranslation (Behr 2018; Behr and Braun 2021). The TRAPD approach through which we translated BESSI from English to German comprised 5 steps: (T) In the translation phase, two independent translators (a translation expert and an expert psychometrician) translated the instruction, items, and response scales of BESSI from English to German. (R) In the review stage, the translators and two independent experts reviewed the two translations and decided on the final translations. Where necessary, they suggested alternative translations. (A) In the adjudication phase, an independent adjudicator who had not been involved in the prior phases compared the different translations against the English source version, chose between competing version in cases in which the reviewers had not agreed on a final translation for an item, and approved the translation for the fieldwork. (P) The pretest phase consisted of presenting the translated instructions, items, response scales to a small number of psychometrics experts and laypersons to test whether all translations were properly understood. The second part of the pretest phase was the initial data collection presented in the following. (D) Finally, the documentation phase consisted in documenting the previous phases in the project’s OSF archive as well as in the present manuscript. We denote the initial translation resulting from the TRAPD approach as BESSI-G v0.1. 

We administered the 192 items of the initial version of BESSI-G (v0.1) to respondents in a three-form planned missingness design (Graham et al. 1996). We randomly assigned respondents to one of three different questionnaires, each of which comprised four out of six items per facet (i.e., 128 out of 192 items) in three different combinations. This design reduced survey length (and hence costs and respondent burden) by one-third. Resulting data are completely missing at random (MCAR) that can be analyzed with standard missing data methods without incurring bias (Zhang and Yu 2021).

#### 4.1.3. Analyses

*Unidimensionality.* Unidimensionality holds if there exists a single latent variable underlying a set of items (Hattie 1985; Ziegler and Hagemann 2015). Only when a scale is unidimensional can scale scores be unambiguously interpreted as reflecting the target skill. Unidimensionality is thus a prerequisite for unbiased estimates of validity and reliability. Moreover, unidimensionality is a prerequisite for using item parcels in later analysis (e.g., Little et al. 2002; Matsunaga 2008), as Soto et al. (2022) did in their analyses of the original BESSI. We tested unidimensionality via the minimum average partial test (MAP; Velicer et al. 2000), parallel analysis (PA; Horn 1965), and the Empirical Kaiser Criterion (EKC; Braeken and van Assen 2017). 

EKC (Braeken and van Assen 2017) uses an eigenvalue decomposition of the inter-item correlation matrix to identify reliable factors. EKC can be seen as a sample-specific variant of the commonly used (population-appropriate) Kaiser-Guttman criterion, which is to retain factors with an eigenvalue greater than 1. However, EKC incorporates random sample variations of the eigenvalues and uses an empirical correction factor before retaining dimensions (Auerswald and Moshagen 2019). MAP (Velicer et al. 2000) identifies the number of systematic components in a correlation matrix through a series of principal component analyses. In each step, components from the preceding step are partialled out. The step number with the lowest average squared partial correlation resulting from the matrices’ off-diagonals (reflecting common variance) indicates the number of components to retain. PA (Horn 1965) contrasts the empirical eigenvalues to those resulting from simulating random data with the same number of variables and observations as the empirical data set. Any factor to be retained must exceed the 95th percentile of the random eigenvalue distribution (Crawford et al. 2010; Zwick and Velicer 1986). As suggested by Lim and Jahng (2019) and Crawford et al. (2010), we used the full correlation matrix and extracted principal components (not factors) to avoid overextraction bias. 

EKC, MAP, and PA offer complementary information for assessing dimensionality. Because their performance varies with the characteristics of the items and scales (i.e., number of items, distribution of responses), we computed all three to obtain an informative picture of the scales’ dimensionality. Although none of them perform better than the other two in all empirical scenarios, we gave EKC the greatest weight because EKC performs as well or better than other indices for relatively short scales such as the six-item BESSI facets (Auerswald and Moshagen 2019; Braeken and van Assen 2017). EKC also performs equally well when the true model is a zero, one, or two-factor-model, which renders it a good choice for testing the unidimensionality of the BESSI-G scales. We provide further details on the three tests in Appendix A, which illustrates why they may diverge and provide complementary information. Additionally, we inspected the first eigenvalue and the ratio of the first to second eigenvalue. Although there can be no universal cutoffs for how large eigenvalues or their ratio should be, larger values are generally preferable.

*Reliability.* AIn line with Soto et al. (2022), we computed two measures of internal reliability: Cronbach’s alpha (α) and McDonald’s omega (ω). Both are measures of the reliability of a unit-weighted scale score; whereas α assumes an at least essentially τ-equivalent model, ω assumes a τ-congeneric model and can be used even if there are correlated errors (Widaman and Revelle 2022; Zinbarg et al. 2006). For that reason, we mainly focused on ω. Additionally, we estimated the highest and lowest possible split-half reliability of each facet resulting from all possible combinations of assigning items to test halves. Moreover, we estimated the test–retest stability over a period of approximately 1.5 months that elapsed between T0 and T2 (median time interval across all respondents: 45 days). We used the pseudo-indicator method (PIM) as described by Rose et al. (2019) to handle missing data with full-information maximum likelihood estimation (FIML).

Reliability depends on scale length and is sample-specific. There are no universally accepted cut-offs for what constitutes sufficient reliability. For individual diagnostic decisions, stricter standards apply than for research purposes. We tentatively judged internal reliability estimates of .60–.70 as “acceptable” and .80 or greater as “very good” (Hulin et al. 2001).

As an ancillary statistic, we computed average variance extracted (AVE), which indicates the share of variance in an item set that can be attributed to the latent construct as opposed to uniqueness and random error (Fornell and Larcker 1981). AVE is therefore often considered to be a measure of factorial validity. Fornell and Larcker (1981) suggested a threshold value of AVE ≥ .50, although lower values are frequently observed. We tentatively adopted the same threshold.

*Factorial validity (CFA measurement models).* To test factorial validity, we fit a single-factor confirmatory factor analysis (CFA) measurement model for each of the 32 facets. The six items per facet were loaded on a single factor whose variance we fixed to unity for identification. These single-factor models test the local independence of the items given the (single) latent trait and will indicate poor fit if local independence is violated. Thus, single-factor CFA models constitute another, arguably strict, test of unidimensionality (Hattie 1985). Moreover, these models inform about the factorial validity of each facet when conceived as a unitary construct. Importantly, even scales that are unidimensional according to the dimensionality tests discussed above may show insufficient fit according to the strict standards of CFA, for example because some items have (residual) correlations beyond the common latent variable that lead to misfit if they go unmodeled (i.e., because local stochastic independence does not always hold).

We estimated all models with a robust maximum likelihood estimator (MLR) and FIML to handle missing data. In line with current conventions for judging model fit (Hu and Bentler 1999), we chiefly relied on the comparative fit index (CFI), root mean square error of approximation (RMSEA), and the standardized root mean square residual (SRMR) to assess model fit. We judged model fit to be acceptable according to the following rules of thumb: CFI > .90 (“adequate”) or > .95 (“good”), RMSEA < .05 (“good”) or at least < .08 (“adequate”), and SRMR < .05 (“good”) or at least < .10 (“adequate”). We stipulated that a model was acceptable when at least two of the three indices passed the cutoffs.

### 4.2. Results

We summarize the main results of the pilot study here. We present the tables with detailed results in Appendix A (Table A1, Table A2 and Table A3).

#### 4.2.1. Unidimensionality of the 32 BESSI-G Facets

Table A1 shows unidimensionality results for the BESSI-G facets. For all 32 facets, there was only one large eigenvalue, whereas the second eigenvalues were small throughout, resulting in ratios of the first to second eigenvalue of 2.53 to 7.20. For 31 of the facets, all indices unequivocally indicated unidimensionality. The sole exception was the self-reflection skill facet, where—despite a clearly dominant first eigenvalue—all tests pointed to a second factor, indicating that the unidimensionality assumption was violated.

#### 4.2.2. Reliabilities of the 32 BESSI-G Facets

Table A2 shows reliability estimates for the BESSI-G facets. For 28 of the 32 facets, ω exceeded .80, the threshold conventionally seen as indicating “good” reliability. The three remaining facets (e.g., Abstract Thinking Skill) fell short of this standard by only a small margin. The average ω across the 32 facets was .85.

Test–retest stabilities (*r*_tt_) of the observed scores over 1.5 months were slightly lower than internal consistencies. They ranged between .66 and .87 with an average of .75. The facet with the lowest test–retest stability in this sample was Impulse Regulation, while that with the highest was Leadership Skill.

AVE was in excess of >.50 for 22 of the 32 facets. The other ten facets fell short of this threshold. For the latter scales, the respective common factor explained only a relatively small amount of variance in the indicators, whereas item uniquenesses/errors were relatively large.

#### 4.2.3. Factorial Validity of the 32 BESSI-G Facets

Table A3 shows the fits of single-factor CFA models for the 32 facets. Although the model χ^2^ indicated significant deviations for all models, many of the facet scales of BESSI-G 0.1 showed satisfactory fit according to conventional cutoffs for at least two of the fit indices. For eight facets, all three fit indices indicated acceptable fit, and for an additional seven facets at least two out of three fit indices signaled acceptable fit. However, fit indices still showed room for improvement for most facets, and nine facets did not achieve good fit in the present sample.

The model modification indices suggested that for most of the insufficiently fitting models, an unmodeled residual covariance for a sole item pair (and only rarely more than one item pair) was responsible for the misfit. That is, these two items were not fully locally statistically independent given the latent variable. Across the 32 facets, the average χ^2^ values of the highest modification index for a residual correlation was 110.02. Upon closer inspection, the reasons for these residual covariances appeared to be trivial in many cases, such as specific words or grammatical constructions that the two items had in common. For example, the first (“Learn about other cultures”) and fourth (“Study other languages or cultures”) item from the cultural competence both referred to learning/acquiring knowledge about other “cultures”. In other cases, the reasons behind the residual correlations with the highest modification indices were less obvious, for example in the case of the teamwork skill items 2 (“Contribute to group projects.”) and 5 (“Cooperate to get things done”).

### 4.3. Discussion

Study 1 demonstrated that the initial version of BESSI-G already achieved satisfactory psychometric properties in the present sample. With the sole exception of Self-Reflection, all facets were clearly unidimensional. The facets’ internal reliabilities were mostly very good (ω ≥ .80 for 28 facets). They were slightly lower than those reported by Soto et al. (2022) for the English-language source version of BESSI, which might be due to the different research design (recall that we applied a three-form design in which each respondent answered different combinations of four out of six items). Test–retest stabilities across a roughly six-week period were lower than internal reliabilities but still moderately high. Overall, these findings suggest that BESSI-G reliably measures a person’s SEB skills.

Despite the facets’ unidimensionality and good reliabilities, single-factor CFA models showed mixed results. Model fit was acceptable for several of the facets according to CFI and SRMR, whereas RMSEA was mostly above the conventional threshold. Generally, the χ^2^ values signaled room for improvement. It should be noted that BESSI was conceived with item parceling in mind, which is why the original publication (Soto et al. 2022) did not test single-factor measurement models for individual BESSI facets. Modification indices suggested that one pair of items per facet was more strongly interrelated than the common factor allowed. Combined with the evidence for unidimensionality and reliability, this suggests that the lack of model fit for most facets was unlikely to reflect major problems with the scales. Moreover, we expected that—if desired—the lack of fit could be remedied by modeling a residual correlation between one item pair—a possibility that we explored in Study 2.

In sum, the initial version of the 32 BESSI-G facet scales already showed promising psychometric properties. However, at least in the present sample, some of the facets showed some room for improvement regarding the fit of single-factor CFA models using single items as input instead of item parcels for joint models as in the original BESSI publication (Soto et al. 2022). We also found a lack of unidimensionality for self-reflection. Reliability was mostly lower than in the English-language source version. Additional analyses (not reported) also identified some items that showed too much overlap with other facets. We therefore drafted revised versions for 31 of the initial 192 items with the aim to further improve the BESSI-G facet scales. Based on ratings by two of the authors (with regard to content validity and translation quality) and a pretest of the alternative translations in a subsample of respondents who were reinterviewed at T2 and T3, we retained 14 of the revised items to replace their respective original items. The decisions are documented in the project’s OSF archive. We thus obtained a refined version of BESSI-G (henceforth “BESSI-G v0.2”) that we fielded in a fresh sample in Study 2.

## 5. Study 2: Testing the Refined BESSI-G Facets and Their Domain-Level Structure

The purpose of Study 2 was threefold. First, we aimed to assess the psychometric properties of the refined version of BESSI-G’s (v0.2) facet scales, repeating the same analyses as in Study 1 in a fresh (and larger) adult sample. 

Second, we expanded our analyses of the factorial validity of BESSI-G over Study 1 by testing its joint facet-level and domain-level structure in addition to the 32 single-factor CFAs. Adopting the same modeling strategy as Soto et al. (2022) with the English-language source version of BESSI, we estimated a joint measurement model with all 32 facets to test the overall facet-level structure of BESSI-G. Moreover, we used exploratory factor analyses (EFA) to test whether the 32 facets cluster in the 5 domains as described by Soto et al. (2022) and Napolitano et al. (2021) for the English-language source version. We expected to replicate the five-dimensional structure of BESSI that these authors reported, including its interstitial skills and the three compound skills (see Figure 1).

Third, we aimed to garner novel insights into the BESSI facets that are of more substantive interest. Next to testing the test–retest stabilities over approximately 8 months—a much longer period than the 1.5-month period in Study 1—we estimated the true score correlations ρ_tt_ (i.e., latent correlations correcting for measurement error) over the same period to gauge the temporal stability of the 32 facets over an extended time period. Moreover, we computed the sample means of the facets to garner insights into the SEB skill distribution in this adult sample.

### 5.1. Method

#### 5.1.1. Data

Data for Study 2 (henceforth “T4”) came from 1008 adults aged 18 to 65 years residing in Germany whose native language was German. We collected the data via a commercial online access panel provider (Respondi AG). Respondents received a small monetary incentive for participation. There was a quota for age, gender, and education according to the German Microcensus 2017, ensuring that the sample was sufficiently diverse and reflected the sociodemographic compositions of the general population. Different from Study 1, about half (n = 517) of the respondents received the full 192-item questionnaire, whereas the other half (n = 491) of the respondents received the same three-form PMD as in Study 1. In this way, information from the full design could be borrowed for handling the missing data introduced by the PMD, adding further precision. After checking data quality and screening out a small number of invalid responses (e.g., straightliners), our final analysis sample for Study 2 comprised 940 respondents.

Incidentally, a subset of 238 of these adults had already participated in the T2 survey of Study 1, in which we had pretested the revised version of BESSI-G. Of those, 203 provided valid data on BESSI-G at both time points. We exploited this overlap in the respondent pool to estimate the test–retest stability of BESSI-G v0.2 over a period of approximately 8 months.

#### 5.1.2. Materials

Respondents answered the 192 items of BESSI (v0.2). Fourteen of these items differed slightly from the earlier version. The instructions and response scale remained identical. The items can be found in Table A6 in Appendix B and in a spreadsheet in the OSF archive at https://osf.io/9pvmj/?view_only=16e79cfced2743aab00d937215a8fe17.

#### 5.1.3. Analyses

Psychometric properties of the 32 skill facets. To test the psychometric properties of the 32 BESSI-G (v0.2) facet scales, we assessed the unidimensionality, reliability, and factorial validity (i.e., CFA model fit) of BESSI’s 32 individual skill facets as described in Study 1. 

In addition to estimating the test–retest correlation of the observed scores, we used the repeated-measures data from T0 and T2 to estimate the true-score correlation ρ_tt_ over 8 months for each facet in a latent-variable framework. The models contained residual correlations across time points between the corresponding items (as required for longitudinal models). We imposed metric invariance over time by fixing the loadings and residual correlation to the same value at both time points. We then extracted the latent (i.e., true-score) correlation between the two time points for each facet.

*Facet-Level and Domain-Level Structure.* To assess the fit of BESSI-G’s facet structure in its entirety, we fit a joint CFA model containing all 32 BESSI-G facets as correlated first-order facets. To ensure comparability with the original BESSI publication, we followed the same analysis strategy as Soto et al. (2022). That is, we used item parceling in order to reduce model complexity, facilitate model convergence, and improve the distributional properties of the manifest indicators. We computed three parcels per facet (96 parcels in total) by assigning each of the 192 items to a two-item parceling the same way as Soto et al. (2022) (i.e., the three parcels consisted of Item 1 and Item 4, Item 2 and Item 5, and Item 3 and Item 6, respectively) and then taking the mean across the two items in each parcel. Given that no cross-loadings are permitted in CFA models, this still constitutes a strict test of the 32 facets’ joint structure. Different from Soto et al. (2022), we used a robust maximum likelihood estimator (MLR) instead of the mean and variance adjusted weighted least squares (WLSMV) estimator to estimate the model. Because the parcel scores followed nearly normal distributions and were quasi-continuous, using WLSMV was not necessary.

To test the domain-level structure of BESSI-G, we conducted an exploratory factor analysis (EFA) with oblique target rotation using the 32 facet scores as input. The target matrix for the rotation contained the theoretical loadings. Following Soto et al. (2022), each facet had a unit loading on its main domain and a zero loading on other domains. The interstitial facets (i.e., Energy Regulation, Information Processing, Ethical Competence, and Impulse Regulation) had loadings of 0.5 on two domains. We did not specify target loadings for the three compound skills (i.e., Adaptability, Capacity for Independence, and Self-Reflection). We then compared how closely the pattern of EFA loadings resembled that of the original BESSI. For this purpose, we first rotated the matrices towards the loading matrix reported by Soto et al. (2022, Table 7) by means of oblique target rotation and then computed the factor congruency (Tucker’s ϕ). Values in the range of .85 ≤ ϕ ≤ .94 indicate “fair” similarity, whereas values in excess of .95 imply that factors can be considered equal (Lorenzo-Seva and Ten Berge 2006).

*Descriptive statistics for the 32 facets.* For each of the 32 BESSI-G facets, we computed the sample mean of the observed scores, that is, unit-weighted mean scores as in Soto et al. (2022), and its standard error to construct 95% confidence intervals. We also computed additional moments (e.g., skewness, kurtosis) for each facet score that we report in Table A5 in Appendix A.

### 5.2. Results

#### 5.2.1. Unidimensionality of the BESSI-G (v0.2) Facets

Table 1 shows the dimensionality results for the Study 2 sample. All three indices were in unison, suggesting that all 32 facets were unidimensional. Compared to BESSI-G v0.1 investigated in Study 1, the first eigenvalues tended to be higher, with an average of 4.29 compared to 3.60 in the previous sample. Consequently, the ratio of the first to the second eigenvalue was larger (8.26 compared to 4.67 in Study 1). Thus, we concluded that unidimensionality held for all BESSI-G (v0.2) facets.

#### 5.2.2. Reliability and Temporal Stability of the BESSI-G (v0.2) Facets

Table 2 shows reliability estimates for the BESSI-G facets. Although reliabilities were already good in Study 1, they further improved in the fresh sample of Study 2. Internal consistencies were now in excess of .80 for 32 of the 32 facets and often surpassed .90. The average ω across the 32 facets was .90 and thus virtually identical to what Soto et al. (2022) obtained in multiple samples with the English-language source version. AVE now passed the threshold of >.50 for 32 of the 32 facets, indicating that the common factors explained more variation per item on average compared to Study 1.

As one would expect, test–retest stabilities (*r*_tt_) over 8 months were lower than those across 1.5 months in Study 1. Recall that test–retest stability reflects measurement error (i.e., unreliability) and trait change as well as state fluctuations. Still, *r*_tt_ ranged between .40 and .80 with an average of .66. The facet with the lowest *r*_tt_ was, somewhat ironically, Capacity for Consistency, which was the only facet with a test–retest correlation below .50.

The true-score stabilities ρ_tt_ (i.e., test–retest correlations corrected for measurement error trough latent-variable modeling) ranged from .69 to .91 with an average of .79. On average, ρ_tt_ exceeded *r*_tt_ by .13, although the difference was often much greater. Hence, the true score stabilities of the skills were all substantial. 

#### 5.2.3. Factorial Validity of the BESSI-G (v0.2) Facets

Table 3 shows the model fits of the 32 single-factor CFAs for BESSI (v0.2). The fit of the 32 single-factor CFAs improved over Study 1 for most of the facets. Still, several of the facets did not fully meet conventional cutoffs—despite now even clearer evidence for their unidimensionality and reliability. Time Management and Self-Reflection Skill showed the poorest fit, whereas other facets such as Responsibility Management showed good fit.

As in Study 1, modification indices suggested that misfit arose from unmodeled residual covariances (i.e., violations of local stochastic independence). Across the 32 facets, the average χ^2^ values of the highest modification index for a residual correlation was 96.03. We therefore tested the fit of measurement models that additionally included one residual covariance for the item pair with the highest modification index. Such residual covariances are likely to reflect similarities in item wording or grammatical constructions that two items share with each other (but not with the remaining four items). Accounting for this misfit by modeling the residual covariances could improve model fit but otherwise leave model interpretation intact. Results shown in Table A4 in Appendix A suggest that most models achieved acceptable fit after introducing one residual correlation. In all cases, model fit improved over the models without the residual correlation, and all but one facet now showed good fit with CFI > .95, SRMR < .05, and in most cases RMSEA < .08. The sole exception exhibiting insufficient fit in the Study 2 sample was the Time Management facet. Because this facet had shown good fit in Study 1 (see Table A3 for BESSI-G v0.1) and the items had remained unchanged in v0.2, we concluded that its poorer fit in Study 2 was likely attributable to sampling variation. Overall, this suggests that the misfit in the measurement models of the BESSI-G facets was mostly trivial, arose from linguistic similarities between some items, did not threaten the overall factorial validity of the model, and could (if desired) mostly be remedied by introducing a single residual covariance. Although further improvements might be possible by introducing a second residual covariance, we did not pursue any further data-driven model modifications but accepted the current measurement models for all facets.

**Figure 2:**
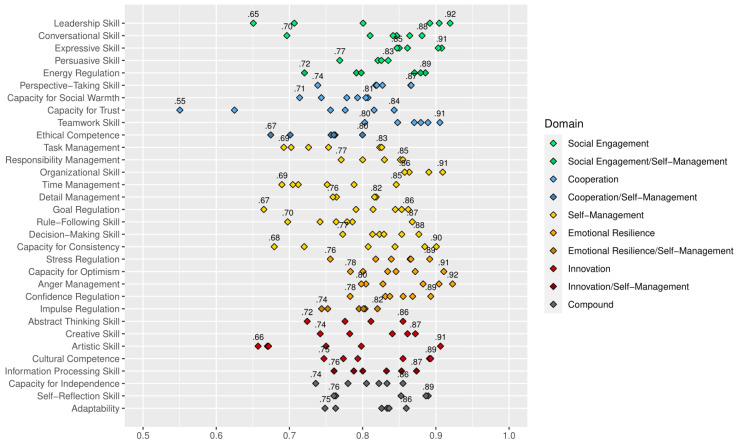
Standardized CFA loadings (λ) of the BESSI-G v0.2 items on their respective factors (Study 2).

All factor loadings in these CFA models were moderate to high, ranging from .54 to .92 with an average of .81. Figure 2 displays the loadings of all items on their respective facet factor based on the improved models shown in Table 3. The figure shows that, in fact, 167 out of 192 standardized loadings (i.e., 86%) were λ ≥ .70, indicating consistently strong relationships between the latent variables and their indicators.

#### 5.2.4. Facet-Level and Domain-Level Structure

*Joint CFA model for the 32 BESSI-G facets.* A joint (i.e., correlated first-order factor) CFA model for the 32 facets showed good fit to the data, χ^2^ (3968) = 6909.03, *p* < .001, CFI = .96, RMSEA = .03 [.03, .03], SRMR = .03. These fit indices are on par or slightly better (especially for RMSEA) than those obtained for the English-language source version in different samples (see Soto et al. 2022, Table 6), although the fits are not directly comparable because these authors used a WLSMV estimator and not MLR.

The standardized loadings of the 96 parcels on their respective factors had a range of .80 ≤ λ ≤ .97 with an average of λ = .90. Because the parcels’ loadings were high and homogeneous, we explored whether a stricter (and more parsimonious) model, namely, an essentially τ-equivalent model in which all three parcels had equal loadings on their respective latent variable, fit the data. The fit of the essentially τ-equivalent joint CFA model was acceptable, χ^2^ (4032) = 7237.17, *p* < .001, CFI = 0.95, RMSEA = 0.03 [0.03, 0.03], SRMR = 0.03. The model also had a better balance of fit and complexity/parsimony (BIC = 113,973.68) compared to the τ-congeneric model (BIC = 114,056.46). Thus, the more parsimonious essentially τ-equivalent should be preferred over the τ-congeneric model.

**Figure 3:**
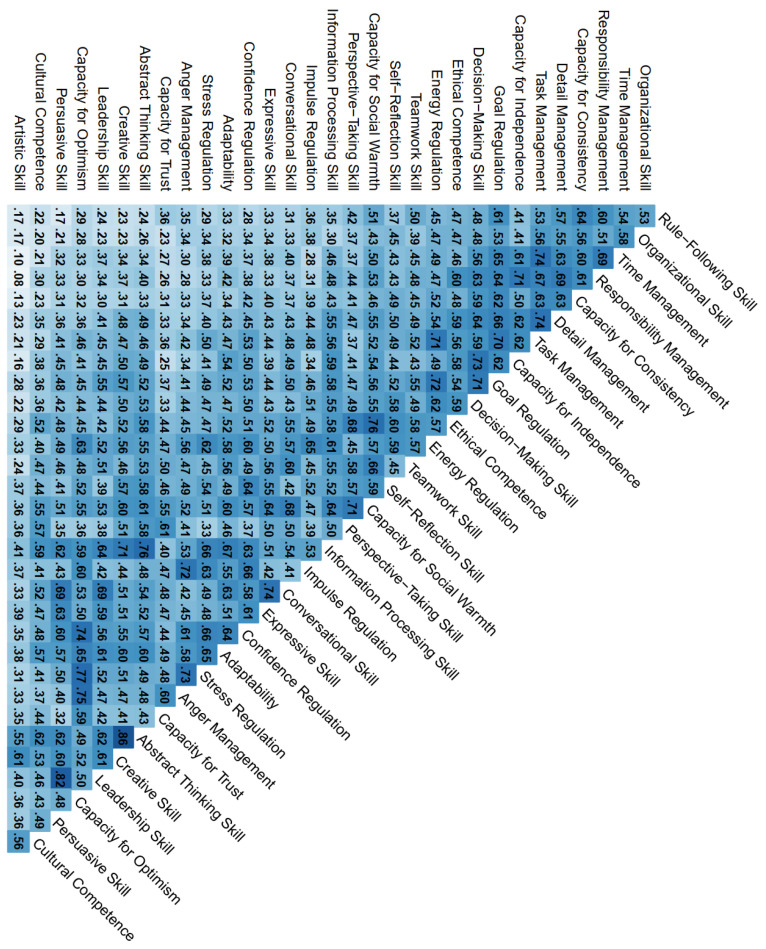
Latent correlations among the 32 BESSI-G (v0.2) facets.

Figure 3 shows the zero-order correlations between the 32 latent variables from the joint CFA model. The skill facets formed a positive manifold, meaning that all correlations among them were positive. The correlations ranged from small (*r* = .08 between Artistic Skill and Responsibility Management) to high (*r* = .86 between the two Innovation Skill facets Creative Skill and Abstract Thinking Skill). The facets’ intercorrelations were approximately normally distributed around their average of $\bar{r}$ = .49. Most fell in the .40 ≤ *r* ≤ .60 range, showing that the BESSI-G facets were related (as one would expect) but at the same time far from redundant. The facet that, on average, had the smallest correlations with all other facets was the Artistic Skill facet ($\bar{r}$ = .32), whereas the facet that, on average, had the strongest correlations with other facets was Capacity for Social Warmth ($\bar{r}$ = .56).

**Figure 4:**
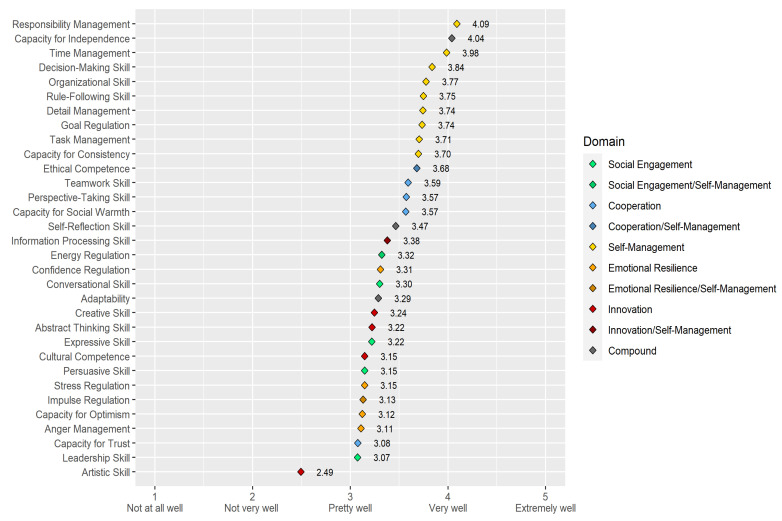
Sample means of the SEB facet scores.

Figure 4 shows the means and 95% confidence intervals of the BESSI-G facets’ observed scores—the type of scores most researchers working with BESSI will be using. The scores are sorted in descending order by their sample mean. The facets are colored by the domain(s) to which they are assigned according to Soto et al. (2022) and Napolitano et al. (2021). Table A5 in Appendix A shows additional descriptive statistics.

Several observations about Figure 4 are noteworthy. First, most means were above the scales’ midpoint of three, indicating that respondents, on average, thought that they mastered these SEB skills “pretty well” to “very well”. This also implies that most observed scores were skewed towards higher skill levels, an impression that is confirmed by the descriptive statistics in Table A5. Second, respondents rated their skills most highly in the Self-Management domain. All facets of this domain were among the ten top-rated skills. Third, the facet with by far the lowest mean was Artistic Skill, which was the only facet whose sample mean was below the scale’s midpoint.

*Domain-level structure of BESSI-G.* Table 4 shows target-rotated loadings from the EFA model testing the domain-level structure of BESSI-G when extracting five factors. Additionally, it shows two indices: “Complexity” refers to the number of factors needed to account for the observed variable (in this case: the facet score). Complexities of 1 would imply a perfect simple structure in which each facet loads on only one factor, whereas complexities greater than one imply that the facet loads on multiple factors. “Uniqueness” refers to the variance that is unique to each facet and not shared with other facets; it equals one minus the communality; for example, a uniqueness of .20 suggests that 20% of a facet’s variance is not shared with any other facets.

Of the 25 skill facets that could be uniquely assigned to exactly one of the five domains according to their loadings by Soto et al. (2022) (see Figure 1), all had their highest loading on the expected domain factor in our present data as well. The overall pattern of loadings was in close alignment with that of the English-language BESSI reported in Soto et al. (2022). The congruency coefficients between their BESSI loading matrix and our present BESSI-G loading matrix ranged between .93 ≤ ϕ ≤ .94 per domain, indicating that the domain factors were highly similar or in fact equivalent when comparing BESSI-G to BESSI. When comparing the loadings against the idealized target matrix containing only 0 and 1 loadings, the congruencies were still quite high (.82 ≤ ϕ ≤ .97), implying a good fit between theoretical expectations and the empirical loading pattern.

Some differences to the English-language source version emerged in the details, specifically, the interstitial and compound skills. Four facets (i.e., Energy Regulation, Information Processing, Ethical Competence, and Impulse Regulation) were labeled as “interstitial” facets by Soto et al. (2022) and Napolitano et al. (2021) because they loaded similarly highly on two domains. In the present sample, the Ethical Competence facet was a truly interstitial facet, loading equally on both Cooperation and Self-Management. The other three primarily loaded on one factor: Energy Regulation on Self-Management (and to a lesser extent Emotional Resilience but not Social Engagement as in the original BESSI), Information Processing Skill on Innovation; and Impulse Regulation on Emotional Resilience). Thus, the “interstitial” facets tended to fall more clearly under a single domain in our sample compared to the original BESSI. On the other hand, Cultural Competence had a non-negligible cross loading on the Cooperation domain.

Regarding the compound skills that did not load on any of the five domains in the original BESSI, two of the three (namely, Adaptability and Self-Reflection Skill) likewise did not have a strong and dominant loading (all λ ≤ .40) on any of the five domains. These facets also had the highest complexities and were thus indeed “compound skills”. By contrast, the Capacity for Independence facet clearly fell under the Self-Management Skills domain—as originally intended by Soto et al. (2022) but different from these authors’ empirical findings in samples from the US. Thus, Adaptability and Self-Reflection but not Capacity for Independence were compound skills.

### 5.3. Discussion

BESSI-G (v0.2) performed well in terms of unidimensionality, reliability, and factorial validity on both the facet and domain level. All facets now were clearly unidimensional, had high to very high internal consistencies comparable with the English-language source version (average ω = .90) and moderate-to-high test–retest correlations across a period of approximately 8 months. With an average true score correlation of ρ_tt_ = .79, the 8-month stabilities (correcting for measurement error) were substantial and in line with the hypothesis that SEB skills are relatively stable over time (though malleable in principle).

The model fits of single-factor CFAs per facet were mostly acceptable, although some facets still did not meet conventional cutoff criteria of model fit (Hu and Bentler 1999). We do not deem the remaining misfit very problematic for three reasons. First, the conventional fixed cutoffs should not be overgeneralized, and there is no need to fully reject a model if it fails to meet the conventional cutoffs (e.g., Groskurth et al. 2021). Second, the sources of misfit seemed to be mostly trivial, arising from shared wording effects. After introducing a single residual correlation per facet to account for wording effects, model fit improved (Table A4). This is a strategy that we do not generally recommend but that researchers may choose to pursue if further optimizing model fit is the goal. Third and most important, it should be kept in mind that Soto et al. (2022) designed BESSI with a joint 32-facet model based on item parcels in mind and that did not optimize the fit of individual facets. It is important to note that moderate amount of model misfit is unlikely to introduce major bias in coefficients of interest (e.g., means, correlations) when using item parcels as input in CFA models, especially since the unidimensionality assumption held for all 32 facets. The same applies when using the observed unit-weighted scale scores for the BESSI facets, which will be the default way in which most users will work with BESSI(-G).

We also replicated the facet-level structure and domain-level structure of BESSI proposed by Soto et al. (2022). A joint CFA facet model for the 32 facets showed good fit to the data. Additional analyses showed that the joint CFA model even fit when applying the restriction that all parcels load equally on their target facet (i.e., an essentially τ-equivalent model). Correlations in the joint CFA model revealed that the 32 facets formed a positive manifold in which most facets were positively correlated, with latent (i.e., true score) correlations ranging from small (*r* < .10) to substantial (*r* ~ .80) with an average slightly below .50. At the same time, the correlations indicated that all facets were sufficiently distinct from all others, offering unique information about a person’s SEB skills.

Moreover, an EFA closely replicated the domain-level structure of the English-language source version of BESSI. All BESSI-G core facets that Soto et al. (2022) could clearly assign to one of the five domains fell under the same domain in our sample. The only differences to the source version we observed were in the finer details, namely, the loadings of the interstitial and compound facets. By and large, however, our findings lend further support to the organization of the 32 BESSI(-G) facets in five global SEB skill domains that resemble the Big Five in the realm of personality traits (Soto et al. 2022) in both English and German.

The descriptive statistics suggested that respondents ascribed to themselves rather high levels on many SEB skills (mostly between “pretty well” and “very well”). Social desirability may be one of the factors behind the relatively high means, an explanation that future studies could test by contrasting self-reports with informant-reports.

## 6. Study 3: Convergent and Discriminant Validity

Having established the final translation of the BESSI-G items in Study 2, the aim of Study 3 was to locate BESSI-G’s facets and domains in a nomological network with the two arguably most important and historically dominant individual difference constructs: personality traits and intelligence. We tested correlations (for some of which we preregistered hypotheses in the project’s OSF archive) between the BESSI-G domains facets, personality domains and facets, as well as fluid and crystallized intelligence.

In line with the original BESSI publication of Soto et al. (2022, Study 4), we tested associations of each BESSI-G facet with the Big Five personality traits. By design, BESSI’s facets share many of the social, emotional, and behavioral referents with the Big Five. Consequently, the domain-level structure of BESSI established by Soto et al. (2022) and Napolitano et al. (2021) resembles the Big Five, which have been the dominant framework for assessing SEB skills (much prior research on SEB skills even used traditional Big Five inventories to measure SEB skills; Lechner et al. (2019)). We therefore sought to replicate the convergent validity of the BESSI domains in relation to the Big Five. At the same time, BESSI intends to measure skill (functional capacities), not traits, leading us to expect that BESSI-G would exhibit discriminant validity against the Big Five. In this regard, we generally expected to find the same patterns of associations that Soto et al. (2022, Study 4) reported for the English-language source version of BESSI. These authors found convergent correlations between the BESSI domains and corresponding Big Five domains ranging from *r* = .67 for Cooperation and Agreeableness to *r* = .79 for Social Engagement and Extraversion. The discriminant correlations of the BESSI domains with the non-corresponding Big Five domains ranged from .09 to .42 in Soto et al. (2022, Study 4).

Moreover, expanding previous evidence on the validity of BESSI, in Study 3 we present first evidence on the associations of the 32 facets with both fluid (*g*_f_) and crystallized intelligence (*g*_c_). Evidence based on Big Five inventories (e.g., the BFI-2 in Rammstedt et al. 2018; the BFI-10 in Lechner et al. 2017) suggests that Big Five personality traits are largely independent from crystallized and especially from fluid intelligence, despite some systematic and replicable associations especially with the Openness domain. Recent results by Guo et al. (2022) based on the OECD’s Study on Social and Emotional Skills (SESS; Chernyshenko et al. 2018), which contains a Big Five-based, faceted measure of SEB skills, closely echo these findings. These authors found that SEB domains and facets had only small to moderate correlations with a short measure of mostly fluid cognitive abilities, the highest associations being those of tolerance (*r* = .17) and curiosity (*r* = .16), two facets from the Open-Mindedness domain (corresponding to BESSI’s Innovation Skills domain). Based on this prior work and for conceptual reasons (i.e., that SEB skills are designed to measure abilities other than intelligence), we expected that SEB skills are largely independent of both fluid and crystallized intelligence, with two important exceptions: we expected the facets from the Innovation skills domain (especially Abstract Thinking Skill and Information Processing Skill, and to a lesser extent Intercultural Competence, Creative Skill, Artistic Skill) to correlate positively with fluid and crystallized intelligence.

### 6.1. Method

#### 6.1.1. Sample

Study 3 used data from the subsample of 767 respondents who participated in the follow-up waves of our data collection in which we assessed intelligence (T1) and piloted BESSI-G v0.2 (T2).

#### 6.1.2. Measures

*BESSI-G.* We used the v0.2 of BESSI-G as evaluated in Study 2, measured at T2.

*Big Five.* We measured the Big Five personality traits with the short Big Five Inventory-2 (BFI-2-S; Soto and John 2017) in its German adaptation (Rammstedt et al. 2020). BFI-2-S measures each Big Five domain with 6 items (i.e., 30 items in total). We administered 15 of the items at T0 and the further 15 items at T2. Respondents rated each item on a five-point scale ranging from 1 (disagree strongly) to 5 (agree strongly). Note that the original BESSI paper by Soto et al. (2022) used the full 60-item BFI-2. The BFI-2-S has only 2 items instead of 4 per facet. Therefore, we expected associations on the facet level to be slightly lower, whereas the differences at the domain level should be negligible.

*Fluid intelligence.* We assessed fluid intelligence (*g*_f_) with 12 items from the International Cognitive Assessment Resource (ICAR; Condon and Revelle 2014), a short measure of intelligence that shows convergent validity with longer standard intelligence measures (e.g., Young and Keith 2020). These items measured 3 subsets: Verbal Reasoning (VR), Letter and Number Series (LN), and Matrix Reasoning (MR). Each set of four items was presented in three separate blocks with a time limit of 2, 3, and 3 min, respectively. Participants could work on block-tasks at their own speed and/or skipping blocks via a progress button. They were required to indicate 1 out of 8 options (1 correct option, 6 distractors, plus “None of these”, or “I don’t know”) for each item. When the time limit was reached, the assessment automatically jumped to the next block. Answers were recoded to 0 for wrong, do not know, or non-given answers and to 1 for the correct solution. The final sum score ranges from 0 to 12. We used data from 607 respondents who took the assessment before 6 February 2021, when we changed the time limit for the assessment as part of a survey experiment for another study unrelated to the present BESSI-G validation. Reliability of the 12-item sum score of ICAR in our sample was α = .73.

*Crystallized intelligence.* To assess *g*_c_, we used the short version of the Berliner Test zur Erfassung fluider und kristalliner Intelligenz (BEFKI GC-K; Schipolowski et al. 2014). BEFKI contains 12 items that cover basic knowledge from humanities, natural, and social sciences. For each item, participants are asked to mark 1 out of 4 possible answers. Following the test’s manual, we limited the assessment time to 5 min. We recoded respondents’ answers to 0 for wrong or missing and to 1 for the correct answer, such that the (number-right) test scores range from 0 to 12. Schipolowski et al. (2014) reported good factorial validity, small to medium correlations with socio-economic and personality measures (e.g., Big Five Openness, *r* = .21), and good reliabilities (Cronbach’s α = .81, or for the manifest sum score, Raykov’s ρ = .70). As with *g*_f_, we used data from 607 respondents. Reliability of the 12-item sum score in our sample was α = .68.

#### 6.1.3. Analyses

Following the original BESSI paper (Soto et al. 2022), we computed Pearson correlations between observed scores to evaluate the nomological network of BESSI-G. For the facet-level correlations, we used the PIM method (Rose et al. 2019), which enabled us to compute observed scores using FIML to account for any missing data arising from our planned missingness design. We estimated separate models for each bivariate correlation between a BESSI-G facet with a Big Five facet, *g*_f_, or *g*_c_. For the domain-level correlations, PIM models did not converge in many cases, such that we decided to use the prorated mean (i.e., the mean across all available items per respondent) for all models involving the domains. Because missing data was fully random, using the prorated mean would not introduce significant bias.

To gauge the similarity of the correlations between BESSI and personality traits in our sample with those reported by Soto et al. (2022), we computed two statistics: (1) the pattern correlations (i.e., the Pearson correlations of the Fisher-*z*-transformed correlations for each column vector in the respective correlation table) and (2) the average absolute difference of the Fisher-z-transformed correlations per column vector. We report these statistics in each correlation table involving the BESSI facets and domains and the Big Five facets and domains.

### 6.2. Results

#### 6.2.1. Associations with Personality Traits

Table 5 shows the correlations between the 32 BESSI-G facets and the Big Five personality traits. The strongest correlations per facet are highlighted in bold. The BESSI-G facets were moderately linked with the Big Five. Few correlations—to be specific, only 15 out of 160—exceeded *r* = .50. Similar to the English-language source version of BESSI, with very few exceptions each BESSI-G facet had at least one (and mostly only one) correlation with a Big Five domain that exceeded *r* = .30. The majority of BESSI-G’s facets had their strongest correlation with the Big Five domain that corresponds to these facets’ BESSI domains.

Table 6 shows associations between the BESSI-G facets and the 15 personality facets of BFI-2-S. Several insights can be gleaned from the table. First, some of the BESSI-G domains had substantial associations on the observed-score level with a personality facet that supported their convergent validity. For example, we observed strong convergent associations (*r* ≥ .60) between Leadership Skills and Assertiveness, Organizational Skill and Organization, Creative Skill and Creative Imagination, as well as Stress Regulation and Anxiety. Second, however, most correlations were not as strong, with only 22 out of the 480 correlations in Table 6 exceeding a value of .50. Apart from typically one or two personality facets that often came from the corresponding Big Five domain, the BESSI-G facets had mostly small associations with personality facets, supporting their discriminant validity. With a few exceptions, the pattern of correlations was similar to that reported by Soto et al. (2022).

Table 7 additionally shows the correlations at the domain level. As expected, each BESSI-G domain had its highest correlation with the Big Five domain to which it corresponds theoretically according to Soto et al. (2022). The correlations with the other four Big Five domains—shown in the off-diagonals of the table—were consistently weaker, such that there was a single substantial correlation per BESSI-G domain. With a maximum value of *r* = .68 and an average of $\bar{r}$ = .58, the convergent correlations in the diagonal of the table were slightly weaker than those reported by Soto et al. (2022), yet the general pattern was highly similar. Thus, as hypothesized, the BESSI-G domains showed convergent validity with the Big Five as well as discriminant validity.

#### 6.2.2. Associations with Intelligence

Table 8 shows the correlations of the 32 BESSI-G facets with fluid (*g*_f_) and crystallized (*g*_c_) intelligence. Most correlations were near zero. None exceeded an absolute value of |*r*| = .20. Thus, in line with our hypotheses, most SEB skills measured by BESSI-G were largely independent of intelligence. The highest correlation of any BESSI facet with *g*_f_ was that of Information Processing Skill at *r* = .20. Facets from the Innovation Skills domain—Information Processing Skill, Abstract Thinking Skill, and Cultural Competence—were among those that had the strongest associations with *g*_f_. Other facets with small positive correlations to *g*_f_ were those from the Self-Management domain: Responsibility Management, Detail Management, and Capacity for Independence, which was a compound facet in the original BESSI (Soto et al. 2022) but fell under the Self-Management domain in Study 2. In turn, facets that loaded on the Self-Management domain—Responsibility Management, Decision-Making Skill, Detail Management, as well as Ethical Competence and Capacity for Independence—were among those that had the highest correlations with *g*_c_. The facet with the strongest association to *g*_c_ was Responsibility Management at *r* = .16. Information processing skill also had a non-negligible positive association with *g*_c_.

Table 9 shows the correlations for the BESSI-G domains. All correlations were close to zero. The largest correlation was that between Innovation Skills and *g*_f_ at *r* = .08.

### 6.3. Discussion

Study 3 demonstrated that BESSI-G’s 32 facets are associated in expected and theoretically plausible ways with personality traits (i.e., Big Five domains and facets as measured with BFI-2-S). The pattern of associations was highly similar to the one Soto et al. (2022, Study 3) observed for the English-language source version of BESSI, although—likely as a result of the lower reliability of BFI-2-S compared to the full BFI-2—the correlations tended to be weaker than in the original study by Soto et al. (2022). This means that BESSI-G closely resembles BESSI not only in how the skill facets related to each other and their higher-level domains (see Study 2) but also in how the skill facets related to personality traits. Notably, although some associations were substantial even on the observed-score level that we investigated here (i.e., not controlling for measurement error and attenuation), few associations were strong enough to suggest complete overlap between personality traits and the SEB skills measured by BESSI-G. Despite the fact that both the Big Five and SEB skills share the same referents, the skill-focused framing and response format of BESSI (asking about a person’s skill levels instead of typical behavioral tendencies that characterize them) achieved sufficient discriminant validity from personality traits.

Moreover, Study 3 was the first to investigate how the SEB skills measured by the BESSI assessment framework relate to intelligence. As expected, most of the 32 BESSI-G facets were largely unrelated to both fluid and crystallized intelligence, at least in the low-stakes situation investigated here. This is similar to what prior research (e.g., Rammstedt et al. 2018; Lechner et al. 2017; Guo et al. 2022) has reported for Big Five domain and facets measures. Also akin to this earlier research, the facets with the strongest relation to intelligence came from the Innovation Skills domain (corresponding to Open-Mindedness in the BFI-2) and, for *g*_c_, those from the Self-Management Skills domain (corresponding to Conscientiousness): Responsibility Management, Detail Management; as well as Cultural Competence and Information Processing Skill, which had the strongest associations with *g*_f_. These findings suggest that only a few BESSI-G facets have—theoretically plausible—links to *g*_f_ and *g*_c_. Correlations were even smaller at the domain level.

These findings give further credence to the view that SEB skills are functional capacities, many of which can be cultivated largely independent of cognitive ability (e.g., Lechner et al. 2017; Rammstedt et al. 2020). A possible alternative explanation that we could not rule out at this stage, however, is that people do not have fully accurate perceptions of their SEB skills, which in turn may limit any associations between their self-reported SEB skills and their—more objectively measured—fluid and crystallized intelligence. In the realm of cognitive ability, meta-analytic correlations between self-perceived and measured ability are often in the vicinity of *r* ≈ .30, although with large variation across types of abilities (Freund and Karsten 2012; Zell and Krizan 2014); it is unclear whether the same might apply to SEB skills, mainly because objective, maximum-performance measures of SEB skills are in short supply. A meta-analysis by McDaniel et al. (2007) that analyzed associations with general intelligence (*g*) of situational judgement tests (SJTs) used for personnel suggestion (including some SJTs that incidentally measure skills somewhat similar to some of the SEB skills measured by BESSI) found somewhat stronger associations between SJTs and g than we did with BESSI, especially when the SJTs used knowledge (*r* = .32) as opposed to behavioral tendencies (*r* = .17) instructions. Thus, future research assessing associations with intelligence using additional measures of BESSI’s facets (such as SJTs and informant-ratings) will be able to provide further insights into how cognitive and SEB skills are related. These studies should also investigate these associations in high-stakes settings.

## 7. General Discussion

In this paper, we presented BESSI-G, a German-language adaptation of the recently introduced BESSI (Soto et al. 2022; see also Napolitano et al. 2021). BESSI-G is the first foreign-language adaptation of BESSI. We expanded the results presented by Soto et al. (2022) on the English-language source version by (1) assessing the psychometric properties of the 32 individual facets (in addition to their joint facet-level and domain-level structure), (2) providing first insights into the temporal stabilities of the SEB skills measured by BESSI (in addition to internal reliabilities), and (3) investigating these facets’ associations with intelligence (in addition to personality traits).

Results from our three studies demonstrate that BESSI-G has good psychometric properties that are in many ways comparable to the English-language source version. BESSI-G’s facets are all unidimensional, have good reliabilities that are high enough even for practical applications, and exhibit mostly acceptable CFA model fit, especially after allowing one item pair per facet to have a residual correlation. The facets also fit well when modeled jointly in a 32-facet CFA with parcels as input; they even conformed to an essentially τ-equivalent model with equal factor loadings for all parcels. Moreover, the facets cluster in the five domains as expected when modeled with an EFA. The organization of the 32 facets in 5 higher-order domains resembling the Big Five was highly similar to the English-language source version and supported the BESSI framework (Napolitano et al. 2021; Soto et al. 2022). The same applied to the patterns of associations with personality traits, which closely resembled those of the source version.

Our findings contribute to the wider debate about how to best conceptualize SEB skills. A large number of frameworks have been proposed (for reviews, see Abrahams et al. 2019; Schoon 2021; Soto et al. 2022). Where many of these frameworks—including BESSI—build on the familiar Big Five framework and hence share many similarities and a common language, others take a more theory-driven approach that does not directly map on the Big Five. Examples include the Values-in-Action (VIA) framework of character strengths and its attendant inventory VIA-IS (Peterson et al. 2005; Ruch et al. 2010), which are based on a cross-cultural analysis of valued traits, and the DOMASEC taxonomy recently proposed by Schoon (2021), which aims to link the Big Five to self-determination theory and other theoretical considerations. Compared to virtually all previous taxonomies and inventories, BESSI has the advantage of allowing for a more fine-grained and comprehensive assessment of SEB skills (e.g., the VIA framework comprises only three global factor-analytic domains; see Partsch et al. 2021) and of unequivocally assessing SEB skills as skills instead of traits or preferences. Despite its recent nature, BESSI rests on a solid psychometric footing and allows for a comprehensive assessment of SEB skills within a relatively short assessment duration. An analysis of time stamps in the Study 2 subsample that answered all 192 BESSI-G items showed that BESSI-G typically took respondents between 10 and 20 min to complete, with an average of about 15 min. This is highly similar to what Soto et al. (2022) reported for the English-language source version and implies that even the relatively long 192-item version—perhaps owing to the simple item wording—might not come with overly high respondent burden. Thus, we believe that BESSI will be a good choice for researchers seeking to investigate SEB skills. That said, future work comparing BESSI(-G) to other SEB skill inventories, especially those not based on the Big Five framework such as VIA, will be helpful in further mapping out the SEB skill space.

Our findings also add to research on the nature of SEB skills and their malleability. The SEB skills measured by BESSI proved to be systematically related (as one would expect) but not interchangeable with personality traits. They also proved to be largely independent of intelligence. Reminiscent of similar findings on personality traits and intelligence (e.g., Lechner et al. 2017; Rammstedt et al. 2018), this suggests that the SEB skills measured by BESSI(-G) are functional capacities that people can develop independent of their highly heritable intelligence. Additionally, our repeated-measures design allowed us to provide first evidence on the test–retest stabilities of the BESSI facets. The facets’ observed scores are moderately stable over a 1.5-month period (average *r*_tt_ = .75) and somewhat less stable over an 8-month period (average *r*_tt_ = .66), yet the temporal stabilities of the true scores across 8 months were substantial throughout (average ρ_tt_ = .79). These temporal stabilities are consistent with the view espoused by many SEB skill researchers (e.g., Abrahams et al. 2019; Kautz et al. 2014; Soto et al. 2022) that SEB skills are relatively stable across the life span, though, in principle, malleable such as through educational experiences and targeted interventions (Brandt et al. 2019; Göllner et al. 2017; e.g., Grosz et al. 2022). BESSI provides a novel instrument that may prove fruitful for future inquiries into the development of SEB skills over the entire life span.

### 7.1. Strengths, Limitations and Directions for Future Research

Our present research has several strengths. It offers an in-depth psychometric analysis in multiple large samples of a comprehensive SEB skill inventory translated through the state-of-art TRAPD approach, including extensive analyses of BESSI’s nomological network with personality traits and intelligence as well as first evidence on the test–retest stability of BESSI’s facets. At the same time, our research has limitations that future research should address.

First, we focused on the self-report version of BESSI-G. For both research and applied purposes, validating an observer-report version would be a natural next step. In the English-language source version, the observer report version of BESSI showed similarly good psychometric properties as the self-report version. Future research should investigate whether—as indeed we expect—the same applies to our German-language adaptation. Going forward, it might also be possible to complement the self- and observer-report forms of BESSI with situational judgement tests (SJTs) or multiple forced choice that might further increase objectivity and reduce social desirability.

Second, we relied on online samples of respondents who answered BESSI-G in the absence of any external pressures (i.e., in low-stakes settings). Future research should investigate whether the psychometric properties of BESSI-G are equally good, or perhaps better, in other survey modes (e.g., paper-pencil) and in high-stakes settings. The latter will be especially important if BESSI-G is to be applied to support placement or admission decisions. In this regard, social desirability and its impact on the validity of BESSI(-G) self-reports will be a crucial issue for future studies to address.

Third, although we investigated the nomological network of BESSI-G’s facets with personality traits and intelligence, we did not yet investigate whether BESSI predicts important outcomes such as success in education, at work, and beyond. Accumulating evidence on its predictive validity will further support BESSI-G’s utility in research and applied settings. Soto et al. (2022) already presented evidence that BESSI predicted a range of criteria, although almost all these criteria were self-reported. Therefore, future research should investigate whether BESSI (including both the self-report and observer-report version) predicts important life outcomes in prospective designs and using non-self-report outcome measures.

Fourth, our samples were confined to adolescents and adults aged 14 to 65 years. Although this is not itself a major limitation, BESSI was developed with an even broader age range in mind. To facilitate developmental research into the precursors, life-span dynamics, and outcomes of SEB skills, it will be important to assess whether BESSI(-G) is equally applicable to children. Given that BESSI(-G) uses short, simple statements, there is reason to be optimistic that the inventory will work in children below the age of 10. However, so far this is only a hope not backed up by evidence. We encourage future studies to test BESSI’s applicability to children.

### 7.2. Conclusions

BESSI-G is a German-language adaptation of BESSI (Soto et al. 2022). It measures 32 SEB skill facets reliably, validly, and efficiently with an average assessment duration of 15 min. These facets cluster in five domains in ways that are theoretically expected and highly similar to the English-language source version. Given its good psychometric properties established in this paper, at this stage, we can recommend BESSI-G (and its English-language source version) for research applications in educational, clinical, developmental, or organizational research. We are hopeful that BESSI-G will enable future research into the assessment and conceptual status of SEB skills, their predictive power for life outcomes, as well as their life-span development (including targeted interventions). BESSI-G is freely available to researchers. Provided that future studies resolve some open questions and limitations that we discussed above, BESSI-G may also become a viable tool for applied contexts, such as SEB skill training and admission or placement decisions.

## Figures and Tables

**Table 1:** Dimensionality of the 32 facets of BESSI-G v0.2 (Study 2).

BESSI Facet	No. of Factors			Eigenvalues		
	PA	MAP	EKC	1st	2nd	Ratio
*Social Engagement Skills*						
Leadership Skill	1	1	1	4.33	0.59	7.38
Conversational Skill	1	1	1	4.39	0.53	8.29
Expressive Skill	1	1	1	4.78	0.42	11.29
Persuasive Skill	1	1	1	4.15	0.68	6.09
Energy Regulation	1	1	1	4.41	0.48	9.11
*Cooperation Skills*						
Perspective-Taking Skill	1	1	1	4.39	0.46	9.46
Capacity for Social Warmth	1	1	1	3.99	0.59	6.75
Capacity for Trust	1	1	1	3.68	0.71	5.20
Teamwork Skill	1	1	1	4.75	0.39	12.09
Ethical Competence	1	1	1	3.76	0.67	5.61
*Self-Management Skills*						
Task Management	1	1	1	3.85	0.62	6.18
Responsibility Management	1	1	1	4.37	0.44	9.89
Organizational Skill	1	1	1	4.89	0.32	15.13
Time Management	1	1	1	3.81	0.96	3.97
Detail Management	1	1	1	4.21	0.50	8.41
Goal Regulation	1	1	1	4.25	0.62	6.84
Rule-Following Skill	1	1	1	3.99	0.65	6.10
Decision-Making Skill	1	1	1	4.43	0.48	9.27
Capacity for Consistency	1	1	1	4.28	0.54	7.97
*Emotional Resilience Skills*						
Stress Regulation	1	1	1	4.53	0.48	9.37
Capacity for Optimism	1	1	1	4.54	0.44	10.29
Anger Management	1	1	1	4.69	0.42	11.06
Confidence Regulation	1	1	1	4.57	0.52	8.84
Impulse Regulation	1	1	1	4.09	0.61	6.72
*Innovation Skills*						
Abstract Thinking Skill	1	1	1	4.16	0.57	7.28
Creative Skill	1	1	1	4.43	0.58	7.65
Artistic Skill	1	1	1	3.79	0.64	5.91
Cultural Competence	1	1	1	4.41	0.53	8.31
Information Processing Skill	1	1	1	4.35	0.48	9.13
*Compound Skills*						
Capacity for Independence	1	1	1	4.25	0.48	8.90
Self-Reflection Skill	1	1	1	4.45	0.59	7.56
Adaptability	1	1	1	4.29	0.51	8.41

**Table 2:** Reliabilities of the 32 facets of BESSI-G v0.2 with 8-month test–retest stabilities and true-score correlations (Study 2).

Facet	α	ω	AVE	Split-Half	$r_{tt}$	$\rho_{tt}$
*Social Engagement Skills*						
Leadership Skill	.92	.91	.66	.90–.94	.80	.91
Conversational Skill	.93	.92	.68	.90–.94	.71	.87
Expressive Skill	.95	.94	.75	.92–.96	.66	.73
Persuasive Skill	.91	.89	.62	.84–.93	.74	.85
Energy Regulation	.93	.92	.68	.91–.95	.70	.80
*Cooperation Skills*						
Perspective-Taking Skill	.93	.92	.67	.91–.94	.64	.82
Capacity for Social Warmth	.90	.88	.59	.87–.93	.66	.78
Capacity for Trust	.87	.83	.52	.81–.91	.74	.82
Teamwork Skill	.95	.94	.74	.93–.96	.67	.71
Ethical Competence	.88	.87	.55	.83–.90	.56	.71
*Self-Management Skills*						
Task Management	.89	.87	.56	.87–.91	.53	.80
Responsibility Management	.92	.92	.67	.91–.94	.61	.69
Organizational Skill	.95	.95	.77	.94–.96	.65	.86
Time Management	.88	.85	.55	.76–.93	.67	.82
Detail Management	.91	.90	.63	.89–.93	.54	.71
Goal Regulation	.92	.89	.63	.86–.93	.68	.80
Rule-Following Skill	.90	.88	.59	.86–.93	.64	.78
Decision-Making Skill	.93	.92	.68	.90–.94	.59	.71
Capacity for Consistency	.92	.93	.67	.88–.94	.40	.71
*Emotional Resilience Skills*						
Stress Regulation	.93	.92	.70	.90–.95	.73	.81
Capacity for Optimism	.94	.92	.69	.92–.95	.77	.82
Anger Management	.94	.93	.73	.91–.96	.65	.77
Confidence Regulation	.94	.92	.70	.90–.95	.74	.79
Impulse Regulation	.91	.89	.61	.86–.93	.56	.74
*Innovation Skills*						
Abstract Thinking Skill	.91	.89	.63	.86–.94	.69	.90
Creative Skill	.93	.91	.67	.90–.95	.71	.82
Artistic Skill	.88	.86	.54	.84–.91	.76	.80
Cultural Competence	.93	.94	.69	.90–.94	.70	.77
Information Processing Skill	.92	.92	.67	.90–.94	.65	.81
*Compound Skills*						
Capacity for Independence	.92	.91	.64	.90–.93	.65	.72
Self-Reflection Skill	.93	.90	.68	.89–.95	.55	.75
Adaptability	.92	.91	.65	.89–.94	.67	.76

**Table 3:** Model fits of the 32 single-factor CFA models for BESSI-G v0.2 (Study 2).

Facet	$\chi^{2}$	*df*	*p*	$\chi^{2}/\mathrm{df}$	CFI	RMSEA	SRMR
*Social Engagement Skills*							
Leadership Skill	43.30	9	*p* < .001	4.81	.985	.070	.022
Conversational Skill	79.16	9	*p* < .001	8.80	.967	.100	.025
Expressive Skill	129.34	9	*p* < .001	14.37	.955	.130	.027
Persuasive Skill	146.73	9	*p* < .001	16.30	.927	.140	.046
Energy Regulation	60.41	9	*p* < .001	6.71	.978	.085	.022
*Cooperation Skills*							
Perspective-Taking Skill	47.56	9	*p* < .001	5.28	.980	.074	.020
Capacity for Social Warmth	86.41	9	*p* < .001	9.60	.952	.105	.033
Capacity for Trust	128.72	9	*p* < .001	14.30	.913	.130	.047
Teamwork Skill	59.82	9	*p* < .001	6.65	.975	.085	.021
Ethical Competence	46.61	9	*p* < .001	5.18	.966	.073	.032
*Self-Management Skills*							
Task Management	67.52	9	*p* < .001	7.50	.965	.091	.031
Responsibility Management	36.34	9	*p* < .001	4.04	.982	.062	.020
Organizational Skill	57.17	9	*p* < .001	6.35	.981	.082	.017
Time Management	330.34	9	*p* < .001	36.70	.810	.212	.081
Detail Management	67.00	9	*p* < .001	7.44	.961	.090	.028
Goal Regulation	125.40	9	*p* < .001	13.93	.937	.128	.039
Rule-Following Skill	111.58	9	*p* < .001	12.40	.944	.120	.037
Decision-Making Skill	63.99	9	*p* < .001	7.11	.972	.088	.025
Capacity for Consistency	46.84	9	*p* < .001	5.20	.975	.073	.027
*Emotional Resilience Skills*							
Stress Regulation	109.66	9	*p* < .001	12.19	.955	.119	.030
Capacity for Optimism	93.13	9	*p* < .001	10.35	.967	.109	.025
Anger Management	123.85	9	*p* < .001	13.76	.939	.127	.032
Confidence Regulation	160.78	9	*p* < .001	17.86	.936	.147	.035
Impulse Regulation	106.92	9	*p* < .001	11.88	.936	.118	.038
*Innovation Skills*							
Abstract Thinking Skill	136.68	9	*p* < .001	15.19	.936	.134	.037
Creative Skill	150.64	9	*p* < .001	16.74	.945	.141	.036
Artistic Skill	97.26	9	*p* < .001	10.81	.946	.112	.039
Cultural Competence	60.89	9	*p* < .001	6.77	.976	.086	.025
Information Processing Skill	71.48	9	*p* < .001	7.94	.967	.094	.026
*Compound Skills*							
Capacity for Independence	66.40	9	*p* < .001	7.38	.965	.090	.027
Self-Reflection Skill	184.58	9	*p* < .001	20.51	.910	.158	.047
Adaptability	115.40	9	*p* < .001	12.82	.945	.123	.032

**Table 4:** EFA loadings for the BESSI-G facet scales.

Facet	Loadings on the BESSI Domains					Indices	
	Social Engagement	Cooperation	Self-Management	Emotional Resilience	Innovation	Complexity	Uniqueness
Conversational Skill	**0.97**	0.31	−0.14	−0.06	−0.23	2.17	.23
Persuasive Skill	**0.88**	−0.17	−0.11	0.01	0.15	2.05	.30
Leadership Skill	**0.78**	−0.14	−0.04	0.04	0.16	2.37	.33
Expressive Skill	**0.69**	0.34	−0.11	0.02	−0.12	3.03	.38
Perspective-Taking Skill	−0.18	**0.85**	0.07	−0.27	0.34	2.06	.26
Capacity for Trust	−0.04	**0.61**	−0.15	0.33	−0.01	2.51	.48
Capacity for Social Warmth	0.21	**0.55**	0.16	−0.07	0.07	3.84	.30
Ethical Competence	−0.02	**0.42**	0.38	−0.11	0.16	3.49	.41
Teamwork Skill	0.28	**0.40**	0.21	0.01	−0.07	4.13	.45
Responsibility Management	−0.07	−0.02	**1.02**	−0.25	−0.06	1.18	.34
Time Management	0.06	−0.11	**0.98**	−0.12	−0.22	1.19	.40
Task Management	0.04	−0.21	**0.93**	0.09	−0.12	1.58	.34
Detail Management	−0.09	−0.02	**0.91**	−0.16	0.07	1.47	.36
Capacity for Consistency	−0.09	0.05	**0.89**	−0.03	−0.20	1.30	.45
Goal Regulation	0.02	−0.12	**0.85**	−0.03	0.07	1.81	.33
Decision-Making Skill	−0.14	−0.05	**0.81**	0.01	0.15	1.89	.40
Capacity for Independence	0.11	−0.17	**0.79**	−0.15	0.12	1.82	.43
Organizational Skill	−0.14	0.13	**0.79**	0.03	−0.26	1.44	.54
Rule-Following Skill	−0.18	0.30	**0.78**	−0.04	−0.27	1.62	.47
Energy Regulation	0.05	−0.04	**0.49**	0.35	0.01	3.09	.38
Capacity for Optimism	0.09	0.01	−0.00	**0.83**	−0.06	1.56	.25
Anger Management	−0.15	0.16	0.03	**0.83**	−0.02	1.49	.29
Stress Regulation	0.05	−0.11	0.16	**0.79**	−0.02	1.67	.29
Impulse Regulation	−0.17	0.05	0.20	**0.65**	0.06	1.84	.43
Confidence Regulation	0.28	−0.08	0.14	**0.53**	0.03	2.77	.35
Abstract Thinking Skill	−0.05	0.04	0.07	−0.09	**0.91**	1.87	.20
Creative Skill	−0.04	−0.07	0.12	−0.01	**0.88**	1.88	.24
Artistic Skill	−0.11	0.17	−0.22	0.05	**0.70**	1.82	.60
Cultural Competence	0.11	0.38	−0.19	−0.05	**0.50**	3.18	.49
Information Processing Skill	0.09	−0.11	0.29	0.15	**0.49**	3.51	.32
Self-Reflection Skill	−0.23	0.23	0.34	0.20	0.29	4.00	.47
Adaptability	0.30	0.04	0.07	0.34	0.13	3.77	.40

*Note*. Loadings with a size of .40 or greater are highlighted in bold.

**Table 5:** Correlations of the BESSI-G facets with the Big Five domains (Study 3).

Facet	Extraversion	Agreeableness	Conscientiousness	Emotional Stability	Open-Mindedness
*Social Engagement Skills*					
Leadership Skill	**.64**	−.08	.19	.22	.18
Conversational Skill	**.60**	.19	.20	.31	.20
Expressive Skill	**.41**	.17	.23	.26	.24
Persuasive Skill	**.53**	−.12	.10	.15	.20
Energy Regulation	.42	.10	.42	**.48**	.21
*Cooperation Skills*					
Perspective-Taking Skill	.17	**.47**	.20	.11	.30
Capacity for Social Warmth	**.44**	.42	.24	.21	.27
Capacity for Trust	.23	**.44**	.00	.30	.14
Teamwork Skill	**.42**	.25	.35	.30	.17
Ethical Competence	.20	.25	**.39**	.28	.23
*Self-Management Skills*					
Task Management	.31	.17	**.57**	.46	.23
Responsibility Management	.22	.28	**.55**	.25	.19
Organizational Skill	.23	.08	**.71**	.19	.07
Time Management	.21	.13	**.56**	.23	.11
Detail Management	.31	.14	**.52**	.25	.34
Goal Regulation	**.46**	.16	.42	.28	.33
Rule-Following Skill	.07	.26	**.37**	.13	.07
Decision-Making Skill	.24	.12	.32	**.34**	.27
Capacity for Consistency	.13	.14	**.39**	.15	.02
*Emotional Resilience Skills*					
Stress Regulation	.18	.06	.13	**.68**	.09
Capacity for Optimism	.37	.27	.22	**.66**	.15
Anger Management	.15	.20	.14	**.59**	.17
Confidence Regulation	.45	.14	.21	**.54**	.18
Impulse Regulation	.15	.19	.26	**.39**	.21
*Innovation Skills*					
Abstract Thinking Skill	.41	.08	.12	.15	**.57**
Creative Skill	.38	.02	.11	.22	**.58**
Artistic Skill	.17	−.03	−.07	−.03	**.53**
Cultural Competence	.29	.18	.07	.08	**.39**
Information Processing Skill	.34	.08	.29	.32	**.36**
*Compound Skills*					
Capacity for Independence	.28	.07	**.40**	.29	.25
Self-Reflection Skill	.30	.20	.25	**.33**	.27
Adaptability	**.45**	.17	.22	.36	.27
Comparison with Soto et al. (2022)					
Pattern correlation	.90	.89	.91	**.93**	.89
Average absolute difference	.10	**.14**	.10	.07	.09

**Table 6:** Correlations of the BESSI-G facets with personality facets of the BFI-2-S (Study 3).

Facet	ex-soc	ex-ass	ex-ene	ag-com	ag-res	ag-tru	co-org	co-pro	co-res	em-anx	em-dep	em-vol	op-aes	op-int	op-cre
*Social Engagement Skills*															
Leadership Skill	.41	**.74**	.26	−.06	−.05	−.05	.14	.17	.14	.22	.25	.04	−.01	.24	.24
Conversational Skill	**.61**	.36	.36	.17	.06	.20	.13	.21	.13	.23	.37	.16	−.01	.22	.30
Expressive Skill	**.40**	.31	.18	.19	.09	.14	.12	.25	.19	.18	.30	.14	.06	.20	.32
Persuasive Skill	.40	**.55**	.21	−.04	−.14	−.09	.05	.14	.06	.18	.19	−.02	−.02	.30	.25
Energy Regulation	.27	.27	.39	.01	.04	.19	.24	**.63**	.28	.44	.47	.28	−.03	.25	.34
*Cooperation Skills*															
Perspective-Taking Skill	.09	.10	.19	**.48**	.38	.25	.10	.13	.29	.02	.09	.16	.18	.26	.25
Capacity for Social Warmth	**.37**	.28	.31	**.37**	.31	.29	.15	.16	.28	.13	.21	.18	.08	.20	.35
Capacity for Trust	.21	.07	.23	.23	.24	**.53**	−.08	.08	.02	.24	.22	.31	.03	.14	.17
Teamwork Skill	.28	.31	**.35**	.21	.18	.21	.21	.30	.28	.23	.29	.20	−.03	.20	.29
Ethical Competence	.10	.15	.17	.22	.20	.20	.20	**.36**	**.36**	.23	.23	.24	.04	.22	.31
*Self-Management Skills*															
Task Management	.12	.26	.30	.06	.12	.23	.30	.61	**.70**	.40	.40	.34	−.01	.26	.33
Responsibility Management	.08	.22	.17	.26	**.63**	.17	.38	.40	.52	.18	.28	.15	.01	.18	.26
Organizational Skill	.15	.13	.22	.03	.15	.01	**.73**	.47	.40	.14	.22	.08	−.03	.04	.19
Time Management	.07	.19	.18	.13	.13	.06	.37	.45	**.50**	.19	.22	.16	−.06	.13	.25
Detail Management	.14	.28	.26	.13	.14	.07	.36	**.43**	.41	.22	.23	.17	.13	.29	.36
Goal Regulation	.24	.39	**.38**	.12	.13	.13	.24	**.38**	.36	.22	.29	.17	.07	.36	.37
Rule-Following Skill	.05	.06	.06	.21	.24	.13	**.29**	**.29**	.26	.10	.12	.11	−.03	.03	.18
Decision-Making Skill	.06	.26	.20	.02	.22	.09	.16	.31	.30	.31	.27	.27	.03	.31	**.32**
Capacity for Consistency	.06	.11	.12	.16	**.60**	.51	.26	.35	.29	.15	.14	.54	−.10	.04	.13
*Emotional Resilience Skills*															
Stress Regulation	.12	.10	.16	−.07	.08	.13	.01	.22	.09	**.70**	.48	.57	−.11	.19	.20
Capacity for Optimism	.27	.19	.34	.10	.22	.31	.06	.25	.21	**.58**	.62	.44	−.08	.21	.28
Anger Management	.11	.06	.14	.03	.17	.27	.02	.20	.13	.50	.40	**.58**	−.01	.26	.19
Confidence Regulation	.35	.33	.32	.05	.10	.19	.10	.25	.16	**.40**	.60	.32	−.06	.21	.32
Impulse Regulation	.05	.11	.17	.08	.20	.18	.15	.29	.20	.31	.29	**.37**	.06	.21	.23
*Innovation Skills*															
Abstract Thinking Skill	.22	.37	.31	.06	.13	.01	.02	.14	.15	.13	.13	.14	.27	**.64**	.44
Creative Skill	.21	.34	.27	−.02	.03	.03	.01	.17	.11	.23	.15	.17	.25	.47	**.64**
Artistic Skill	.18	.10	.11	−.10	.02	.03	−.06	−.01	−.07	.01	−.07	−.02	**.51**	.30	.33
Cultural Competence	.15	.22	.28	.08	.18	.16	.06	.03	.10	.08	.07	.05	.25	**.37**	.28
Information Processing Skill	.16	.34	.24	.04	.11	.06	.15	.33	.23	.34	.26	.23	.08	.39	**.41**
*Compound Skills*															
Capacity for Independence	.10	.31	.20	.00	.14	.04	.26	.32	.36	.29	.29	.14	.02	.27	**.33**
Self-Reflection Skill	.20	.23	.23	.18	.19	.11	.15	.19	.25	.24	**.34**	.22	.04	.34	.26
Adaptability	**.35**	.29	.33	.10	.11	.18	.15	.21	.14	.33	.33	.24	.04	.31	.33
Comparison with Soto et al. (2022)															
Pattern correlation	.94	.90	.74	.79	.60	.70	.92	.91	.79	.88	.91	.81	.89	.88	.77
Average absolute difference	.09	.10	.10	.14	.15	.09	.09	.09	.11	.10	.07	.08	.11	.07	.11

*Note.* Strongest correlation of each BESSI-G facet in bold. The Big Five domains and facets are abbreviated as follows: **ex**traversion—**soc**iability, **ass**ertiveness, and **ene**rgy level; **ag**reeableness—**com**passion, **res**pectfulness, and **tru**st; **co**nscientiousness—**org**anization, **pro**ductiveness, and **res**ponsibility: **em**otional stability—**anx**iety, **dep**ression, and **vol**atility; **op**en-mindedness—**aes**thetic sensitivity, **int**ellectual curiosity, and **cre**ative imagination.

**Table 7:** Correlations of the BESSI-G domains with the Big Five domains (Study 3).

Domain	Conscientiousness	Extraversion	Agreeableness	Emo. Stability	Open-Mindedness
Self-Management Skills	**.61**	.25	.18	.32	.21
Social Engagement Skills	.18	**.60**	.00	.27	.21
Cooperation Skills	.21	.33	**.43**	.29	.23
Emotional Resilience Skills	.17	.29	.13	**.68**	.14
Innovation Skills	.06	.33	.02	.10	**.53**
Comparison with Soto et al. (2022)					
Pattern correlation	**.99**	.99	.99	.99	.97
Average absolute difference	.09	.13	**.24**	.06	.15

**Table 8:** Correlations of the BESSI-G facets with cognitive ability (Study 3).

BESSI-G Facet	Fluid Intelligence (*g*_f_)	Crystallized Intelligence (*g*_c_)
*Social Engagement Skills*		
Leadership Skill	.08	.02
Conversational Skill	−.04	.03
Expressive Skill	−.05	.00
Persuasive Skill	.03	.08
Energy Regulation	−.04	−.04
*Cooperation Skills*		
Perspective-Taking Skill	.05	−.02
Capacity for Social Warmth	.04	.01
Capacity for Trust	.01	−.11
Teamwork Skill	.09	.01
Ethical Competence	.07	.16
*Self-Management Skills*		
Task Management	−.04	.04
Responsibility Management	.12	.16
Organizational Skill	−.08	−.07
Time Management	.01	.06
Detail Management	.12	.15
Goal Regulation	.09	.02
Rule-Following Skill	.07	−.03
Decision-Making Skill	.09	.07
Capacity for Consistency	.02	.11
*Emotional Resilience Skills*		
Stress Regulation	−.01	.04
Capacity for Optimism	−.03	.02
Anger Management	−.01	.00
Confidence Regulation	−.05	.00
Impulse Regulation	.03	−.05
*Innovation Skills*		
Abstract Thinking Skill	.13	.12
Creative Skill	.06	.07
Artistic Skill	.04	−.02
Cultural Competence	.15	.06
Information Processing Skill	.20	.11
*Compound Skills*		
Capacity for Independence	.07	.14
Self-Reflection Skill	−.02	−.01
Adaptability	.05	.00

**Table 9:** Correlations of the BESSI-G domains with cognitive ability (Study 3).

BESSI-G Domain	Fluid Intelligence (*g*_f_)	Crystallized Intelligence (*g*_c_)
Self-Management Skills	.05	.07
Social Engagement Skills	−.02	−.02
Cooperation Skills	.01	−.07
Emotional Resilience Skills	−.04	−.01
Innovation Skills	.08	.01

## Data Availability

All study materials including the BESSI-G items and the documentation of the translation process, can be found in the project’s OSF archive at https://osf.io/9pvmj/?view_only=16e79cfced2743aab00d937215a8fe17. The complete analysis code is available in the first author’s GitLab archive at https://git.gesis.org/lechnecs/bessi-g. We used R (Version 4.1.2; R Core Team 2021) for all our analyses. The final translations are also shown in Appendix B.

## Appendix A

**Table A1:** Dimensionality of the 32 facets of BESSI-G v0.1 (Study 1).

BESSI Facet	No. of Factors			Eigenvalues		
	PA	MAP	EKC	1st	2nd	Ratio
*Social Engagement Skills*						
Leadership Skill	1	1	1	3.72	0.74	5.02
Conversational Skill	1	1	1	4.03	0.72	5.63
Expressive Skill	1	1	1	3.92	0.68	5.77
Persuasive Skill	1	1	1	3.58	0.71	5.03
Energy Regulation	1	1	1	3.59	0.79	4.56
*Cooperation Skills*						
Perspective-Taking Skill	1	1	1	3.54	0.76	4.65
Capacity for Social Warmth	1	1	1	3.24	0.76	4.29
Capacity for Trust	1	1	1	3.13	0.81	3.85
Teamwork Skill	1	1	1	3.87	0.85	4.56
Ethical Competence	1	1	1	3.09	0.90	3.42
*Self-Management Skills*						
Task Management	1	1	1	3.71	0.64	5.76
Responsibility Management	1	1	1	3.86	0.67	5.79
Organizational Skill	1	1	1	3.96	0.84	4.72
Time Management	1	1	1	3.63	0.82	4.41
Detail Management	1	1	1	3.44	0.82	4.19
Goal Regulation	1	1	1	3.73	0.72	5.20
Rule-Following Skill	1	1	1	3.58	0.86	4.16
Decision-Making Skill	1	1	1	3.52	0.73	4.82
Capacity for Consistency	1	1	1	3.54	0.94	3.77
*Emotional Resilience Skills*						
Stress Regulation	1	1	1	3.74	0.73	5.16
Capacity for Optimism	1	1	1	4.04	0.56	7.20
Anger Management	1	1	1	3.95	0.67	5.92
Confidence Regulation	1	1	1	4.06	0.73	5.59
Impulse Regulation	1	1	1	3.34	0.79	4.21
*Innovation Skills*						
Abstract Thinking Skill	1	1	1	2.87	0.99	2.89
Creative Skill	1	1	1	3.62	0.64	5.62
Artistic Skill	1	1	1	3.42	0.98	3.48
Cultural Competence	1	1	1	3.86	0.89	4.35
Information Processing Skill	1	1	1	3.57	0.75	4.76
*Compound Skills*						
Capacity for Independence	1	1	1	3.64	0.76	4.80
Self-Reflection Skill	2	2	2	3.15	1.25	2.53
Adaptability	1	1	1	3.33	0.96	3.47

**Table A2:** Reliabilities of the 32 facets of BESSI-G v0.1 with 1.5 months test–retest reliabilities (Study 1).

Facet	α	ω	AVE	Split-Half	$r_{tt}$
*Social Engagement Skills*					
Leadership Skill	.87	.90	.57	.85–.90	.87
Conversational Skill	.90	.89	.60	.86–.93	.81
Expressive Skill	.89	.88	.59	.85–.94	.72
Persuasive Skill	.86	.89	.54	.83–.89	.78
Energy Regulation	.86	.85	.52	.83–.92	.76
*Cooperation Skills*					
Perspective-Taking Skill	.86	.84	.52	.80–.91	.78
Capacity for Social Warmth	.83	.82	.45	.79–.87	.74
Capacity for Trust	.81	.79	.42	.74–.87	.77
Teamwork Skill	.89	.87	.59	.80–.94	.71
Ethical Competence	.81	.76	.40	.73–.86	.67
*Self-Management Skills*					
Task Management	.88	.85	.53	.84–.91	.78
Responsibility Management	.89	.87	.56	.83–.93	.68
Organizational Skill	.89	.89	.62	.86–.93	.82
Time Management	.87	.91	.55	.81–.91	.78
Detail Management	.85	.82	.48	.79–.89	.68
Goal Regulation	.88	.88	.55	.84–.91	.75
Rule-Following Skill	.86	.84	.50	.79–.92	.73
Decision-Making Skill	.86	.85	.50	.82–.90	.69
Capacity for Consistency	.86	.83	.50	.80–.92	.68
*Emotional Resilience Skills*					
Stress Regulation	.88	.86	.55	.84–.93	.77
Capacity for Optimism	.90	.88	.60	.88–.94	.78
Anger Management	.90	.88	.59	.84–.94	.73
Confidence Regulation	.90	.88	.60	.86–.94	.77
Impulse Regulation	.84	.82	.46	.79–.90	.66
*Innovation Skills*					
Abstract Thinking Skill	.78	.75	.37	.67–.85	.81
Creative Skill	.87	.89	.54	.84–.90	.76
Artistic Skill	.85	.82	.49	.71–.90	.79
Cultural Competence	.89	.86	.56	.83–.94	.77
Information Processing Skill	.86	.84	.50	.81–.91	.75
*Compound Skills*					
Capacity for Independence	.87	.84	.52	.80–.93	.68
Self-Reflection Skill	.82	.78	.43	.62–.90	.67
Adaptability	.84	.81	.47	.73–.90	.71

**Table A3:** Model fits of the 32 single-factor CFA models for BESSI-G v0.1 (Study 1).

Facet	$\chi^{2}$	*df*	*p*	$\chi^{2}/\mathrm{df}$	CFI	RMSEA	SRMR
*Social Engagement Skills*							
Leadership Skill	39.06	s	*p* < .001	4.34	.980	.054	.036
Conversational Skill	62.82	9	*p* < .001	6.98	.964	.073	.048
Expressive Skill	77.06	9	*p* < .001	8.56	.957	.082	.039
Persuasive Skill	57.17	9	*p* < .001	6.35	.961	.069	.039
Energy Regulation	206.82	9	*p* < .001	22.98	.876	.139	.060
*Cooperation Skills*							
Perspective-Taking Skill	109.47	9	*p* < .001	12.16	.923	.099	.052
Capacity for Social Warmth	54.67	9	*p* < .001	6.07	.955	.067	.038
Capacity for Trust	67.19	9	*p* < .001	7.47	.935	.076	.053
Teamwork Skill	242.94	9	*p* < .001	26.99	.859	.151	.084
Ethical Competence	104.84	9	*p* < .001	11.65	.903	.097	.076
*Self-Management Skills*							
Task Management	80.83	9	*p* < .001	8.98	.949	.084	.055
Responsibility Management	125.46	9	*p* < .001	13.94	.917	.107	.064
Organizational Skill	126.03	9	*p* < .001	14.00	.932	.107	.049
Time Management	138.13	9	*p* < .001	15.35	.900	.112	.063
Detail Management	134.97	9	*p* < .001	15.00	.907	.111	.062
Goal Regulation	38.40	9	*p* < .001	4.27	.974	.054	.041
Rule-Following Skill	135.51	9	*p* < .001	15.06	.901	.111	.065
Decision-Making Skill	97.94	9	*p* < .001	10.88	.926	.093	.049
Capacity for Consistency	207.84	9	*p* < .001	23.09	.851	.140	.082
*Emotional Resilience Skills*							
Stress Regulation	152.79	9	*p* < .001	16.98	.906	.119	.057
Capacity for Optimism	118.70	9	*p* < .001	13.19	.933	.104	.050
Anger Management	128.93	9	*p* < .001	14.33	.925	.108	.052
Confidence Regulation	147.35	9	*p* < .001	16.37	.916	.116	.053
Impulse Regulation	95.13	9	*p* < .001	10.57	.922	.092	.052
*Innovation Skills*							
Abstract Thinking Skill	96.63	9	*p* < .001	10.74	.887	.093	.073
Creative Skill	43.82	9	*p* < .001	4.87	.971	.058	.032
Artistic Skill	128.47	9	*p* < .001	14.27	.911	.108	.076
Cultural Competence	292.51	9	*p* < .001	32.50	.849	.167	.074
Information Processing Skill	92.46	9	*p* < .001	10.27	.928	.090	.056
*Compound Skills*							
Capacity for Independence	166.96	9	*p* < .001	18.55	.884	.124	.063
Self-Reflection Skill	200.77	9	*p* < .001	22.31	.815	.137	.117
Adaptability	195.10	9	*p* < .001	21.68	.849	.135	.075

**Table A4:** Fits of the CFA models for BESSI-G v0.2 with one residual correlation per facet (Study 2).

Facet	$\chi^{2}$	*df*	*p*	$\chi^{2}/\mathrm{df}$	CFI	RMSEA	SRMR
*Social Engagement Skills*							
Leadership Skill	12.73	8	*p* < .010	1.59	.998	.027	.009
Conversational Skill	42.51	8	*p* < .001	5.31	.984	.074	.018
Expressive Skill	60.63	8	*p* < .001	7.58	.980	.091	.021
Persuasive Skill	90.65	8	*p* < .001	11.33	.957	.115	.038
Energy Regulation	41.86	8	*p* < .001	5.23	.986	.073	.016
*Cooperation Skills*							
Perspective-Taking Skill	28.18	8	*p* < .001	3.52	.989	.057	.016
Capacity for Social Warmth	40.46	8	*p* < .001	5.06	.980	.072	.023
Capacity for Trust	39.47	8	*p* < .001	4.93	.978	.071	.028
Teamwork Skill	33.34	8	*p* < .001	4.17	.988	.063	.016
Ethical Competence	32.43	8	*p* < .001	4.05	.979	.062	.027
*Self-Management Skills*							
Task Management	27.14	8	*p* < .001	3.39	.989	.055	.017
Responsibility Management	22.04	8	*p* < .001	2.75	.991	.047	.016
Organizational Skill	29.39	8	*p* < .001	3.67	.992	.058	.012
Time Management	179.33	8	*p* < .001	22.42	.908	.165	.060
Detail Management	37.77	8	*p* < .001	4.72	.980	.069	.021
Goal Regulation	64.58	8	*p* < .001	8.07	.970	.095	.036
Rule-Following Skill	53.76	8	*p* < .001	6.72	.976	.085	.023
Decision-Making Skill	41.34	8	*p* < .001	5.17	.983	.073	.022
Capacity for Consistency	26.66	8	*p* < .001	3.33	.988	.054	.023
*Emotional Resilience Skills*							
Stress Regulation	54.53	8	*p* < .001	6.82	.980	.086	.021
Capacity for Optimism	29.87	8	*p* < .001	3.73	.992	.059	.013
Anger Management	56.68	8	*p* < .001	7.08	.975	.088	.020
Confidence Regulation	46.22	8	*p* < .001	5.78	.984	.078	.019
Impulse Regulation	43.45	8	*p* < .001	5.43	.977	.075	.026
*Innovation Skills*							
Abstract Thinking Skill	68.67	8	*p* < .001	8.58	.970	.098	.031
Creative Skill	38.11	8	*p* < .001	4.76	.988	.069	.017
Artistic Skill	41.32	8	*p* < .001	5.16	.980	.073	.025
Cultural Competence	29.35	8	*p* < .001	3.67	.989	.058	.019
Information Processing Skill	49.54	8	*p* < .001	6.19	.978	.081	.022
*Compound Skills*							
Capacity for Independence	38.59	8	*p* < .001	4.82	.982	.070	.020
Self-Reflection Skill	74.46	8	*p* < .001	9.31	.969	.103	.031
Adaptability	50.90	8	*p* < .001	6.36	.979	.083	.022

*Note.* All models contained one residual correlation.

**Table A5:** Descriptive statistics for the observed facet scores (Study 2).

Facet	*N*	*M*	*SD*	*Mdn*	Skewness	Kurtosis
*Social Engagement Skills*						
Leadership Skill	786	3.07	0.91	3.00	−0.09	−0.54
Conversational Skill	786	3.30	0.85	3.17	−0.05	−0.22
Expressive Skill	786	3.22	0.92	3.00	−0.10	−0.38
Persuasive Skill	786	3.15	0.84	3.17	−0.20	−0.30
Energy Regulation	786	3.32	0.78	3.33	0.09	−0.16
*Cooperation Skills*						
Perspective-Taking Skill	786	3.57	0.76	3.67	−0.17	0.01
Capacity for Social Warmth	786	3.57	0.71	3.50	0.05	0.00
Capacity for Trust	786	3.08	0.78	3.00	0.09	−0.21
Teamwork Skill	786	3.59	0.80	3.67	−0.38	0.12
Ethical Competence	786	3.68	0.69	3.67	−0.09	0.09
*Self-Management Skills*						
Task Management	791	3.71	0.69	3.67	−0.27	0.26
Responsibility Management	791	4.09	0.66	4.00	−0.55	0.26
Organizational Skill	791	3.77	0.89	3.83	−0.39	−0.38
Time Management	791	3.98	0.72	4.00	−0.59	0.13
Detail Management	791	3.74	0.69	3.83	−0.23	0.22
Goal Regulation	791	3.74	0.76	3.83	−0.24	−0.28
Rule-Following Skill	791	3.75	0.69	3.83	−0.18	−0.12
Decision-Making Skill	791	3.84	0.74	4.00	−0.26	−0.33
Capacity for Consistency	791	3.70	0.73	3.67	−0.13	−0.16
*Emotional Resilience Skills*						
Stress Regulation	785	3.15	0.89	3.17	−0.09	−0.30
Capacity for Optimism	785	3.12	0.87	3.00	0.00	−0.49
Anger Management	785	3.11	0.87	3.00	0.06	−0.18
Confidence Regulation	785	3.31	0.84	3.33	−0.16	−0.03
Impulse Regulation	785	3.13	0.79	3.00	0.05	−0.05
*Innovation Skills*						
Abstract Thinking Skill	786	3.22	0.84	3.17	−0.10	0.00
Creative Skill	786	3.24	0.89	3.33	−0.14	−0.23
Artistic Skill	785	2.49	0.93	2.33	0.32	−0.60
Cultural Competence	785	3.15	0.89	3.17	−0.22	−0.11
Information Processing Skill	785	3.38	0.76	3.33	−0.10	0.07
*Compound Skills*						
Capacity for Independence	791	4.04	0.70	4.00	−0.44	−0.16
Self-Reflection Skill	785	3.47	0.81	3.50	−0.12	−0.06
Adaptability	786	3.29	0.80	3.17	−0.02	−0.05

## Appendix B

**Instructions**

The translated instructions read as follows:
*Hier findest Du eine Liste von Tätigkeiten oder Dingen, die man tun kann. Bitte wähle jeweils eine Antwort aus, die am besten beschreibt, wie gut Du persönlich diese Tätigkeit beherrschst. Zum Beispiel: “Wie gut kannst Du Arbeitsanweisungen folgen?”. Bitte beachte, dass es nicht darum geht, wie oft oder wie gern Du etwas tust, sondern wie gut Du es kannst.*

**Response Format**

The translated response scale read as follows. Respondents are only shown the verbal labels.

1 = *überhaupt nicht gut*
2 = *nicht so gut*
3 = *recht gut*
4 = *sehr gut*
5 = *extrem gut*

**Items**

Items read as shown in Table A6.

**Table A6:** English-language BESSI source versions (from Soto et al. 2022) and final German translations (BESSI-G v0.2) of the 192 items.

BESSI Domain and Facet	Item Wording—English Source	Item Wording—German Adaptation	Item #
*Social Engagement Skills*			
Leadership Skill	Lead a group of people.	Eine Gruppe führen.	1
	Make decisions for a group of people.	Entscheidungen für eine Gruppe treffen.	33
	Assert myself as a leader.	Die Führung übernehmen.	65
	Take charge of a situation.	Verantwortung übernehmen.	97
	Give a speech.	Eine Rede halten.	129
	Convince people to follow my lead.	Andere davon überzeugen, mir zu folgen.	161
Conversational Skill	Introduce myself to strangers.	Mich fremden Leuten vorstellen.	25
	Meet new people.	Neue Leute kennenlernen.	57
	Make conversation with a stranger.	Mich mit einem Fremden unterhalten.	89
	Talk to people.	Mich mit Leuten unterhalten.	121
	Start a conversation.	Ein Gespräch beginnen.	153
	Talk to classmates or coworkers.	Mit Mitschülerinnen oder Kollegeninnen reden.	185
Expressive Skill	Explain what I am thinking and feeling.	Erklären, was ich denke und fühle.	17
	Express myself.	Mich selbst ausdrücken, mich erklären.	49
	Express my thoughts and feelings.	Meine Gedanken und Gefühle ausdrücken.	81
	Tell people how I am feeling.	Anderen mitteilen, wie ich mich fühle.	113
	Tell people about my emotions.	Anderen gegenüber meine Gefühle zeigen.	145
	Explain what’s on my mind.	Erklären, was mir durch den Kopf geht.	177
Persuasive Skill	Win debates with other people.	In Diskussionen die Oberhand behalten.	13
	Confront people when I disagree with them.	Anderen widersprechen, wenn ich nicht deren Meinung bin.	45
	Change people’s minds.	Andere überzeugen, ihre Meinung zu ändern.	77
	Speak up when I disagree with others.	Das Wort ergreifen, wenn ich anderer Meinung bin.	109
	Win arguments.	Streitgespräche gewinnen.	141
	Be blunt and direct with people.	Anderen offen und direkt meine Meinung sagen.	173
Energy Regulation	Use my energy in productive ways.	Meine Energie sinnvoll nutzen.	7
	Find the energy to get things done.	Mich aufraffen, Dinge anzupacken.	39
	Keep going, even when I’m tired.	Weitermachen, auch wenn ich erschöpft bin.	71
	Maintain a high energy level.	Meine Energie aufrecht erhalten.	103
	Stay active.	Aktiv und voller Tatendrang bleiben.	135
	Keep myself motivated.	Meine Motivation aufrecht erhalten.	167
*Cooperation Skills*			
Perspective-Taking Skill	Sympathize with other people’s feelings.	Mich in andere Menschen hineinversetzen.	2
	Feel compassion for other people.	Mitgefühl für andere empfinden.	34
	Take another person’s perspective.	Die Sichtweise von anderen nachzuvollziehen.	66
	Respect people’s feelings.	Die Gefühle anderer Menschen respektieren.	98
	Sense other people’s needs.	Ein Gespür für die Bedürfnisse anderer haben.	130
	Understand how other people feel.	Verstehen, wie andere Menschen sich fühlen.	162
Capacity for Social Warmth	Make people smile.	Menschen zum Lächeln bringen.	14
	Make people feel comfortable.	Dafür sorgen, dass andere Menschen sich wohlfühlen.	46
	Get along with people.	Gut mit anderen Menschen auskommen.	78
	Make a positive impression on people.	Einen guten Eindruck hinterlassen.	110
	Show people that I like them.	Menschen zeigen, dass ich sie mag.	142
	Put people at ease.	Andere Menschen beruhigen.	174
Capacity for Trust	Let go of a grudge.	Nicht nachtragend sein.	8
	Let people borrow my things.	Anderen meine Sachen ausleihen.	40
	See the good in people.	Das Gute im Menschen sehen.	72
	Assume the best about people.	Nur das Beste von anderen denken.	104
	Forgive people quickly.	Anderen Menschen leicht verzeihen.	136
	Trust people.	Anderen Menschen vertrauen.	168
Teamwork Skill	Work as part of a group.	Im Team mit anderen zusammenarbeiten.	23
	Contribute to group projects.	Etwas zu Gruppenprojekten beitragen.	55
	Work with people toward a shared goal.	Mit anderen Menschen auf ein gemeinsames Ziel hinarbeiten.	87
	Collaborate with classmates or coworkers.	Mit Mitschülerinnen oder Kollegeninnen zusammenarbeiten.	119
	Cooperate to get things done.	Zusammenarbeiten, damit etwas fertiggestellt wird.	151
	Cooperate with other people.	Mit anderen zusammenarbeiten.	183
Ethical Competence	Do what’s morally right, even when it’s difficult.	Das moralisch Richtige tun, auch wenn es schwer fällt.	29
	Take responsibility when I’ve made a mistake.	Verantwortung übernehmen, wenn ich einen Fehler gemacht habe.	61
	Tell the truth, even when I don’t want to.	Die Wahrheit sagen, auch wenn es mir schwerfällt.	93
	Stop myself from lying or cheating.	Mich selbst vom Lügen oder Schummeln abhalten.	125
	Follow my ethical principles.	Meinen eigenen Werten treu bleiben.	157
	Be honest with people.	Ehrlich zu anderen sein.	189
*Self-Management Skills*			
Task Management	Keep working until a task is finished.	An Aufgaben dranbleiben, bis sie abgeschlossen sind.	12
	Get started on tasks.	Aufgaben angehen.	44
	Focus on my work.	Mich ganz auf meine Arbeit konzentrieren.	76
	Keep myself from getting distracted.	Mich nicht ablenken lassen.	108
	Work efficiently, without wasting time.	Effizient arbeiten, ohne zu trödeln.	140
	Concentrate on a task.	Mich auf eine Aufgabe konzentrieren.	172
Responsibility Management	Have other people rely on me.	Mich so verhalten, dass Andere sich auf mich verlassen können.	21
	Follow through on commitments.	Mich an Vereinbarungen halten.	53
	Manage my responsibilities.	Meine Verantwortung wahrnehmen.	85
	Fulfill my duties and obligations.	Meine Pflichten erfüllen.	117
	Keep track of my promises and commitments.	Meine Zusagen und Verpflichtungen im Blick behalten.	149
	Follow through on promises.	Meine Versprechen halten.	181
Organizational Skill	Tidy up after myself.	Meine Sachen aufräumen.	6
	Organize my personal spaces.	Meinen Arbeitsplatz in Ordnung halten.	38
	Keep things neat and tidy.	Dinge sauber und ordentlich halten.	70
	Keep things in order.	Ordnung halten.	102
	Put things back in their proper place.	Dinge zurück an ihren Platz räumen.	134
	Clean up after making a mess.	Saubermachen, wenn ich Unordnung verursacht habe.	166
Time Management	Show up for things on time.	Pünktlich sein.	3
	Get to appointments on time.	Termine einhalten.	35
	Follow a schedule.	Einen Zeitplan einhalten.	67
	Manage my time.	Meine Zeit einteilen.	99
	Organize my schedule.	Meinen Terminkalender organisieren.	131
	Plan out my time.	Mir einen Zeitplan erstellen.	163
Detail Management	Check work for mistakes.	Meine Arbeit auf Fehler überprüfen.	15
	Pay attention to details.	Sorgfältig und detailgenau arbeiten.	47
	Take care of details.	Alle Feinheiten genau beachten.	79
	Find and correct mistakes.	Fehler finden und korrigieren.	111
	Double-check my work.	Meine Arbeit nochmals überprüfen.	143
	Pay careful attention to my work.	Genau und sorgfältig auf meine Arbeit achten.	175
Goal Regulation	Set clear goals.	Klare Ziele setzen.	24
	Make plans to achieve a goal.	Planen, wie ich mein Ziel erreichen kann.	56
	Focus on my most important goals.	Mich auf meine wichtigsten Ziele konzentrieren.	88
	Work hard to succeed.	Hart arbeiten, um Erfolg zu haben.	120
	Work toward my goals.	Auf meine Ziele hinarbeiten.	152
	Set high standards for myself.	Hohe Ansprüche an mich selbst haben.	184
Rule-Following Skill	Do as I’m told.	Tun, was man mir sagt.	18
	Obey the law.	Gesetze befolgen.	50
	Follow instructions.	Anweisungen befolgen.	82
	Do what I’m supposed to do.	Tun, was von mir erwartet wird.	114
	Respect authority.	Autorität respektieren.	146
	Follow the rules.	Mich an Regeln halten.	178
Decision-Making Skill	Make careful decisions.	Wohlüberlegte Entscheidungen treffen.	27
	Stop and think things through.	Mir die Zeit nehmen, Dinge zu durchdenken.	59
	Weigh pros and cons before making a decision.	Vor einer Entscheidung die Vor- und Nachteile abwägen.	91
	Think before acting.	Erst denken, dann handeln.	123
	Think things through carefully.	Dinge sorgfältig abwägen.	155
	Consider the consequences of my decisions.	Die Folgen meiner Entscheidungen bedenken.	187
Capacity for Consistency	Repeat a task consistently.	Eine Aufgabe immer auf dieselbe Weise erledigen.	9
	Keep doing a task, even if it’s boring.	An einer Aufgabe dranbleiben, auch wenn sie langweilig ist.	41
	Follow a consistent routine.	Gleichbleibende Abläufe einhalten.	73
	Repeat a standard procedure many times.	Denselben Arbeitsablauf viele Male wiederholen.	105
	Do the same task over and over again.	Dieselbe Aufgabe immer und immer wieder tun.	137
	Do tasks that are routine or repetitive.	Routinemäßige oder wiederkehrende Aufgaben erledigen.	169
*Emotional Resilience Skills*			
Stress Regulation	Stay calm in stressful situations.	In stressigen Situationen ruhig bleiben.	5
	Stop myself from worrying.	Mich wieder beruhigen, wenn ich Sorgen habe.	37
	Cope with stress.	Mit Stress umgehen können.	69
	Relax when I’m feeling tense.	Mich entspannen, wenn ich gestresst bin.	101
	Calm down when I’m feeling anxious.	Mich beruhigen, wenn ich Angst habe.	133
	Settle down when I’m feeling nervous.	Mich beruhigen, wenn ich nervös bin.	165
Capacity for Optimism	Stop myself from feeling pessimistic.	Keine pessimistischen Gedanken zulassen.	11
	Look on the bright side of things.	Immer das Positive sehen.	43
	Stay in a good mood.	Gut gelaunt bleiben.	75
	Stay positive when something bad happens.	Den Mut nicht verlieren, wenn etwas Schlimmes passiert.	107
	Keep a positive attitude.	Eine positive Einstellung behalten.	139
	Stay optimistic when things go wrong.	Optimistisch bleiben, wenn etwas schiefläuft.	171
Anger Management	Calm down when I’m feeling angry.	Mich beruhigen, wenn ich wütend bin.	20
	Control my temper.	Meine Gefühle im Griff haben.	52
	Control my anger.	Meine Wut in den Griff bekommen.	84
	Stop myself from getting angry.	Mich selbst davon abhalten, wütend zu werden.	116
	Stop myself from getting mad.	Mich selbst davon abhalten, sauer zu werden.	148
	Settle down when I’m feeling annoyed.	Mich beruhigen, wenn ich verärgert bin.	180
Confidence Regulation	Find things to like about myself.	Dinge finden, die ich an mir selbst mag.	26
	Have confidence in myself.	Selbstvertrauen haben.	58
	Find reasons to feel good about myself.	Gründe finden, um mich selbst gut zu finden.	90
	Respect myself.	Mich selbst respektieren.	122
	See my strengths.	Meine eigenen Stärken kennen.	154
	See my good qualities.	Meine positiven Eigenschaften erkennen.	186
Impulse Regulation	Control my cravings.	Meine Verlangen oder Gelüste zügeln.	30
	Resist temptations.	Versuchungen widerstehen.	62
	Break my bad habits.	Schlechte Gewohnheiten ablegen.	94
	Control my impulses.	Mich selbst beherrschen.	126
	Stop myself from acting on impulse.	Mich beherrschen, nicht impulsiv zu handeln.	158
	Avoid temptation.	Versuchungen aus dem Weg gehen.	190
*Innovation Skills*			
Abstract Thinking Skill	Understand abstract ideas.	Abstrakte Ideen verstehen.	4
	Have intellectual or philosophical discussions.	Intellektuelle oder philosophische Diskussionen führen.	36
	Discuss complicated topics and ideas.	Komplizierte Themen und Ideen diskutieren.	68
	Think about the nature of the world.	Darüber nachdenken, wie die Welt funktioniert.	100
	Think deeply about things.	Tiefgründig über Dinge nachdenken.	132
	Feel curious about ideas.	Neugierig und ideenhungrig sein.	164
Creative Skill	Find new ways to do things.	Kreative neue Lösungen finden.	16
	Put ideas together in a new way.	Ideen auf ungewöhnliche Weise verbinden.	48
	Use my imagination.	Meine Vorstellungskraft nutzen.	80
	Come up with creative ideas.	Kreative Ideen entwickeln.	112
	Invent things.	Dinge erfinden.	144
	Come up with new ideas.	Neue Ideen entwickeln.	176
Artistic Skill	Draw or paint.	Malen oder Zeichnen.	28
	Create art.	Kunst schaffen.	60
	Appreciate art, music, or literature.	Mich für Kunst, Musik oder Literatur begeistern.	92
	Create beautiful things.	Schöne Dinge gestalten.	124
	Make music.	Musik machen.	156
	Write stories or poems.	Geschichten oder Gedichte schreiben.	188
Cultural Competence	Learn about other cultures.	Etwas über andere Kulturen lernen.	32
	Understand people from different backgrounds.	Menschen unterschiedlicher Herkunft verstehen.	64
	Appreciate different cultures.	Unterschiedliche Kulturen wertschätzen.	96
	Study other languages or cultures.	Mir Fremdsprachen oder Wissen über andere Kulturen aneignen.	128
	Understand people’s cultural identities.	Kulturelle Eigenheiten anderer Menschen verstehen.	160
	Get along with people from different backgrounds.	Mit Menschen unterschiedlicher Herkunft zurechtkommen.	192
Information Processing Skill	Solve puzzles.	Knifflige Probleme lösen.	22
	Handle a lot of information.	Viele Infos gleichzeitig erfassen.	54
	Make sense of complex information.	Komplexe Informationen verstehen.	86
	Process new information.	Neue Informationen verarbeiten.	118
	Learn things quickly.	Schnell dazulernen.	150
	Find logical solutions to problems.	Angemessene Lösungen für Probleme finden.	182
*Compound Skills*			
Capacity for Independence	Do things independently.	Dinge selbstständig tun.	31
	Think for myself.	Eigenständig denken.	63
	Make decisions on my own.	Entscheidungen selbstständig treffen.	95
	Do things on my own.	Dinge alleine tun.	127
	Make my own choices.	Meine eigenen Entscheidungen treffen.	159
	Get things done by myself.	Dinge ohne fremde Hilfe schaffen.	191
Self-Reflection Skill	Look inside myself.	In mich gehen, in mich hineinhören.	10
	Understand myself.	Mich selbst verstehen.	42
	Understand my emotions.	Meine Gefühle verstehen.	74
	Reflect on my life.	Über mein Leben nachdenken.	106
	Pay attention to my thoughts and feelings.	Auf meine Gedanken und Gefühle achten.	138
	Examine myself and my life.	Über mich selbst und mein Leben nachdenken.	170
Adaptability	Try new things.	Neue Dinge ausprobieren.	19
	Adapt to new surroundings.	Mich an eine neue Umgebung anpassen.	51
	Adjust to new routines.	Mich an neue Abläufe anpassen.	83
	Step out of my comfort zone.	Meine Komfortzone verlassen.	115
	Try something that’s unfamiliar.	Etwas Neues und Unbekanntes ausprobieren.	147
	Adapt to change.	Mit Veränderungen zurechtkommen.	179

*Note.* The rightmost column shows the item number (questionnaire order) according to Soto et al. (2022).
